# Genomic and epidemiological identification of *Pseudomonas aeruginosa* transmission chains and in hospital ICUs

**DOI:** 10.1186/s13059-026-04005-1

**Published:** 2026-02-20

**Authors:** Lucia Graña-Miraglia, Xiaoyi Hu, Cheryl Volling, Laura Mataseje, Michael R. Mulvey, Allison McGeer, David S. Guttman, the Toronto Invasive Bacterial Diseases Network (TIBDN), S. Anceva-Sami, B. L. Coleman, A. J. Jamal, J. Johnstone, A. Li, V. Mahesh, R. Melano, A. Paterson, M. Pejkovska, A. Sultana, T. Vikulova, M. Downing, S. Hota, K. Katz, J. A. Leis, M. Muller, S. Nayani, S. Patel, D. Ricciuto, Z. Zhong

**Affiliations:** 1 Department of Cell and Systems Biology, University of Toronto, Toronto, Canada; 2 Centre for the Analysis of Genome Evolution & Function, University of Toronto, Toronto, Canada; 3 Department of Microbiology, Sinai Health, Toronto, Canada; 4 National Microbiology Laboratory, Public Health Agency of Canada, Winnipeg, Canada

**Keywords:** *Pseudomonas aeruginosa*, Epidemiology, Genome sequence, Hospital ICUs, Strain transmission

## Abstract

**Background:**

*Pseudomonas aeruginosa* (PA) is an opportunistic pathogen that poses a significant threat to hospitalized patients, particularly in intensive care units (ICUs) due to its ability to contaminate the environment and subsequently infect vulnerable patients. This study seeks to demonstrate that genomic data is an accessible, rapid, and robust genomic epidemiological tool that can be used in a clinical or diagnostic setting to improve our understanding of PA transmission dynamics and enhance infection prevention and control strategies.

**Results:**

We performed analysis on 2046 PA isolates recovered from patients and sink drains in seven hospital ICUs. We find that a pairwise genetic distance threshold of 24 SNPs best approximates the maximum expected divergence between clonal strains. The pairing of this genetic threshold and the ELS identifies 82 transmission clusters, encompassing 730 isolates, suggesting both environment-patient and patient-patient transmission. Our analysis identifies PA reservoirs in ICU sink drains that last nearly two years and transmission clusters that span multiple ICUs, indicating inter-hospital transmission or shared external reservoirs. Epidemiological linkage is identified for over 70% of genetically linked environment-patient strain pairs and 49% of genetically linked patient-patient strain pairs.

**Conclusions:**

We find strong support for the use of a genetic threshold to identify epidemiologically linked strains. We find genomic evidence for persistent environmental reservoirs as sources of ICU-associated PA infection. This work underscores the importance of environmental monitoring and infection control measures to mitigate the risk posed by contaminated sinks and other environmental sources.

**Supplementary Information:**

The online version contains supplementary material available at 10.1186/s13059-026-04005-1.

## Background

*Pseudomonas aeruginosa* (PA) is a common environmental bacterium and a particularly adept opportunistic human pathogen. It is one of the leading causes of hospital-acquired bacteremia, pneumonia, urinary tract and wound infections, and a leading cause of mortality for individuals who are immunocompromised or suffer from diseases that impact the lung such as cystic fibrosis, bronchiectasis, and chronic obstructive pulmonary disease [1–6]. The CANWARD study of bacterial infections in Canadian intensive care units (ICUs) from 2007–2016 found that PA was the third most common bacterial pathogen isolated from patient infections [7]. PA’s success as an opportunistic pathogen is partially due to its high rate of antimicrobial resistance and adaptability [8, 9]. The threat of multidrug-resistant PA is so significant that it has been listed as a “Serious Threat” by the United States Center for Disease Control and Prevention [10], a high-priority “ESKAPE” pathogen by the Infectious Diseases Society of America [11, 12], and a “High Priority” pathogen on the World Health Organization list for increased research and development of new antibiotics [13].

PA’s ability to adapt to a wide range of environments and acquire resistance by multiple mechanisms facilitate its colonization and persistence in many moist hospital environments, such as sink drains, faucets, faucet aerators, and respiratory equipment [6, 14–20]. Similarly, PA has been shown to persist on dry floors for five weeks, in aerosols for a few hours, and on fabrics for over a month [20–23]. The environmental persistence of PA creates the risk of exposure among some of the most vulnerable hospitalized patients, especially in ICUs [2, 18, 24–27]. While PA is not a member of the healthy human gut microbiota, it can be found in roughly 10% of healthy individuals and has been reported to have a prevalence up to 50% in hospitalized individuals [9, 28–32]. These gut populations are recognized as important reservoirs for systemic sepsis and secondary infections among critically ill patients [33–35].

One limitation to a greater understanding of PA population biology is the lack of standardized, high-resolution methods for assessing strain transmission. While approaches such as multilocus sequence typing (MLST) [36, 37] and its single nucleotide polymorphism (SNP) based successor, multilocus sequence analysis (MLSA) [38], as well as a variety of older techniques, have been essential for classifying clonal lineages and uncovering broader patterns of transmission, they lack the genetic resolution for tracking very recent transmission events. The recent development of core genome MLST (cgMLST) has significantly improved genetic resolution by focusing on SNPs distributed across the entire core genome [39]. In addition to a substantial gain in resolution, cgMLST shares the same positive features of MLST: it is a standardized, allele-based method that is portable and highly reproducible, allowing for reliable comparisons across laboratories and studies. It allows for automated workflows and readily interpretable primary data. And, finally, it requires relatively inexpensive protocols and data generation platforms that are already widespread in both academic and clinical settings. These features make it especially suitable for routine surveillance and epidemiological investigations ([40], Siddall, 2025 #21933). Despite these benefits, the use of cgMLST does face some challenges. Analytical tools required for the comparative analysis of cgMLST data can still be bioinformatically challenging to implement, particularly when using open-source tools which may require advanced command-line skills and familiarity with custom pipelines [41]. Additionally, although cgMLST favors portability, different cgMLST schemes can still be found across research groups and databases. Nevertheless, these variations in scheme composition typically do not affect the resolution or overall conclusions of comparative analyses, as the core discriminatory power is retained across most implementations [40]. Finally, as the name suggests, cgMLST is restricted to the core genome, defined as the set of genomic regions shared by all strains under study. Consequently, cgMLST does not capture the diversity present in the accessory genome, which is often under weaker selective constraints and harbors much of the genomic variability [39].

High resolution typing methods and full genome sequencing are now being used to identify transmission events and clusters, i.e., transmission pairs that chain together. This work is essential for identifying pathogen reservoirs, transmission events, and most crucially, the causes of contamination. A key component of these studies is identifying which pathogen isolates are members of the same clonal lineage. It is important to emphasize that the usage of ‘clone’ here describes a population of very closely related strains that have evolved from a common ancestor. Prior work from our group used a subset of the data presented in this study, as well as a different analysis method estimated a threshold of 20 SNPs for clonemates [15]. Weimann and colleagues [5] used a reference-based method to identify a threshold of 26 SNPs. Romano-Bertrand and colleagues [42] recently reported a 25 SNP threshold in a retrospective study of 14 PA outbreaks at the Lausanne University Hospital (Switzerland) over a 15-year period. In the same study, they also reviewed published 19 outbreak studies and found a consensus threshold of 15–25 SNPs [42]. Finally, Stribling and colleagues [43] found an average pairwise distance of 38 SNPs between ST-621 isolates from a single hospital and an average distance of 24 and 31 SNPs for two subclones.

In this study, we aimed to assess the utility of PA genomic data as an epidemiological tool to identify recent patient-to-patient or environment-to-patient transmission events in ICUs. We specifically focused on accessible, rapid, and robust genomic epidemiological approaches that have the potential to be used in a clinical or diagnostic setting to improve our understanding of PA transmission dynamics and enhance infection prevention and control strategies. In doing so, we established a genetic (i.e., SNP) threshold to define clonal lineages associated with recent transmission events between patients or between patients and the ICU environment (i.e., sinks). We evaluated these genomic analyses against an epidemiological score derived from anonymized patient records and isolate sampling data. Ultimately, the genomic data revealed transmission events and chains, as well as probable infection reservoirs in ICUs in the Greater Toronto Area.

## Results

### Environmental surveillance and the clinical burden of *P. aeruginosa*

The samples used in this study are part of a randomized controlled trial of standard versus copper sink drains conducted in seven ICUs in the Great Toronto Area from 2017 to 2019 [15]. Environmental sampling was conducted between December 2017 and February 2019, and included the sink drain tailpiece, faucet, and air at a distance of approximately 20 cm from the stream of running water. Environmental sampling was initiated prior to patient enrollment to establish a baseline level of PA contamination within ICU settings. This approach allowed for the identification of existing environmental reservoirs and enabled the distinction between pre-existing contamination and potential patient-derived contamination introduced during the study period. By characterizing the ICU environment before patient exposure, this design enabled us to more accurately attribute potential transmission pathways (e.g., environment-to-patient) and assess the impact of targeted interventions on environmental burden.

Between June 2018 and May 2019, a total of 4,263 patient admissions were recorded, of which 910 were screened for PA. Among those screened, 819 (90%) underwent screening at the time of admission, while 91 (10%) had no admission swab available. Of the admission swabs, 18% tested positive, indicating prior colonization or infection, whereas 72% were negative. Clinical samples, typically collected upon the onset of symptoms, yielded 215 (23%) PA-positive results. Among these, 75 patients had tested negative on admission, 89 had been positive, and for 47 patients, no admission swab was available, making it impossible to determine whether colonization or infection was present prior to hospitalization (Additional file 1: Fig. S1A). During this period, 72 *P. aeruginosa* healthcare-associated infections (PA-HAIs—ICU acquired/associated infection meeting National Healthcare Safety Network (NHSN) criteria [44]) were identified in 60 patients (1.4%). Ten patients experienced two PA-HAIs, one patient had three, while no patient met the criteria for PA-HAI during more than one admission. The median age of patients with PA-HAI was 68.5 years (IQR 60–81), and 55% (33/60) were male (14). Pneumonia accounted for the majority of infections (45/72, 62.5%), with 71% (32/45) classified as ventilator-associated. Within 30 days of infection, 24 patients (40%) died; PA-HAI was considered the primary cause of death in 2 cases (3.3%) and a contributing factor in an additional 20 (33%) (Additional file 1: Fig. S1B). Among the 60 patients with PA-HAI, 22 (36.7%) were colonized with *PA* on admission, 28 (46.7%) were not colonized, and colonization status was unknown for 10 patients (14).

### Characterization of sequenced *P. aeruginosa* isolates: source distribution and phylogenetic diversity

**Fig. 1:**
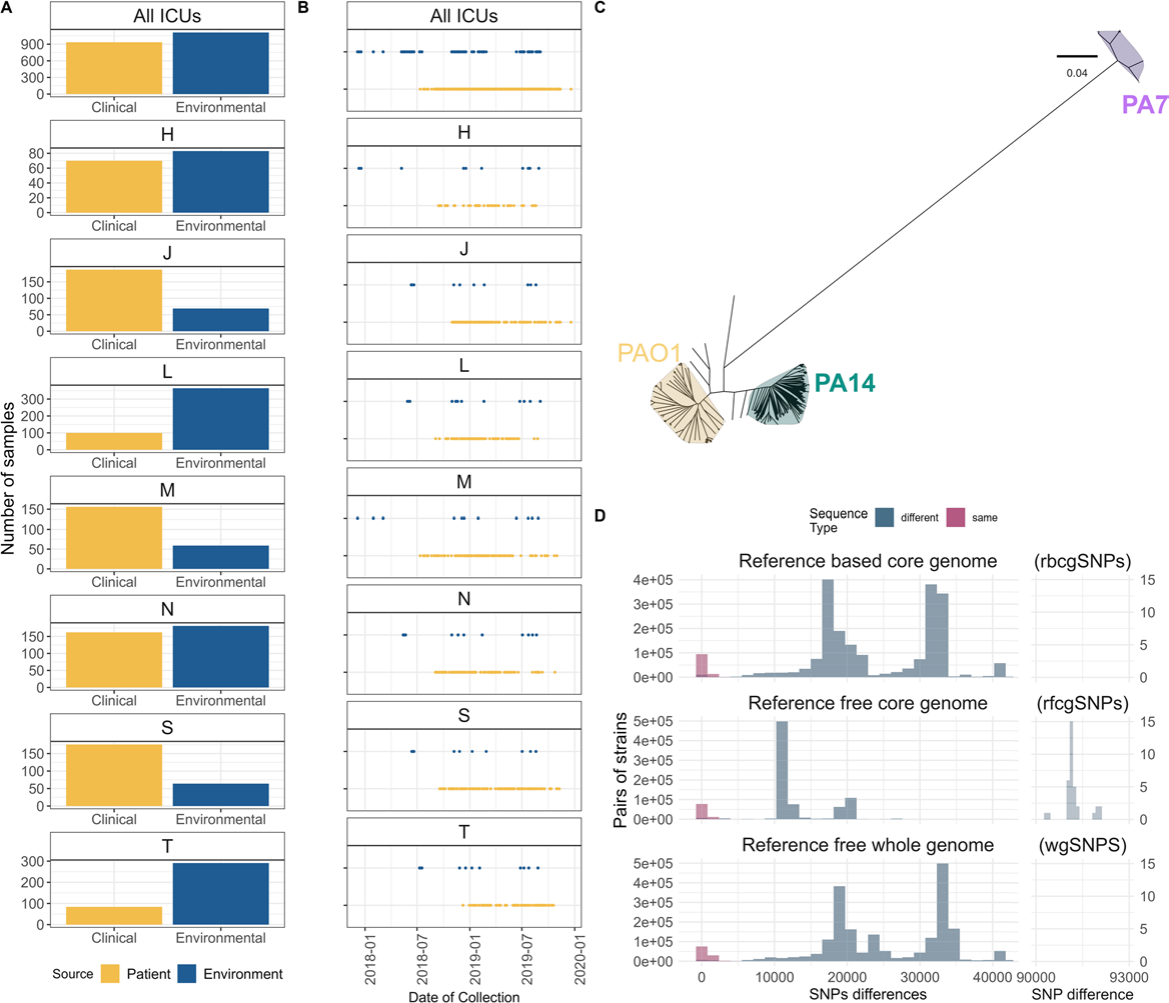
**A** The number of isolates sampled from patients (yellow) and from the ICU environment (blue) in total and for each of the seven hospital ICU in the study. **B** Sampling time span for patient and environmental samples, with blue indicating environmental islates and yellow indicating patient isolates. **C** Maximum likelihood core genome phylogenetic reconstruction including three reference genomes (PAO1, PA14 and PA7) that allowed us to identify the three major lineages of PA species present in our database. **D** Histograms comparing the pairwise SNPs identified in the three different approaches. Red indicates comparisons of strains within an MLST sequence type (ST), while grey indicates between pairs of isolates from different MLST STs. The y-axis represents the frequency of occurrence, while the x-axis denotes the pairwise SNPs count. The reference free core genome approach (middle panel) shows the most extreme values above 90,000 SNPs differences but with a very low frequency

We sequenced PA positive clinical isolates, isolates from the screening of surveillance rectal swabs to identify colonization with PA, and environmental isolates from ICU sink surfaces and drains, air proximal to sinks, and faucets. Isolation source, patient code, ICU, and collection date are available for all isolates, while more detailed information, such as room and admission date, are only available for a subset of the samples. The total number of PA isolates, as well as the proportion recovered from infected patients and the ICU environment, differ between ICUs (Fig. 1A). These differences don’t necessarily reflect variations in the incidence of PA in the ICUs; as these ICUs have differing bed numbers and occupancy rates, and the numbers of sinks randomized in the study differed in different units.

Two thousand and forty-six isolates passed the quality control standards for read number and draft assemblies completeness. Of these, 935 were isolates from patient specimens (hereinafter clinical isolates) and 1111 were isolates from environmental sampling. Clinical isolates were from 374 ICU admissions in 359 patients (some patients had multiple ICU admissions during the study). Eighty-three rectal swabs that were obtained upon ICU admission from 53 individual patients tested positive for PA, indicating colonization prior to ICU admission. Overall, 172 patients had a single PA isolate from specimens obtained during their ICU stay(s), while 202 patients had multiple PA isolates (median 2, range 2–30) for a total of 763 additional isolates.

The majority of isolates were assigned to 157 sequence types (STs), with only a minority (122, 5.9%) of isolates corresponding to newly identified, previously unknown STs. The most frequent sequence types reported were ST 253 (12.9%), ST 179 (11.1%), and ST 175 (10%). High-risk epidemic clones such as ST 235 and ST 244 were also identified but comprised fewer than 2% of isolates. A significant proportion of isolates (1265, 62%), both clinical (443) and environmental (822), were assigned to 20 of the 21 predominant sequence types (STs) in the global epidemiological clones, which are responsible for 51% of clinical PA infections worldwide [5]. The prevalence of different sequence types differed significantly between ICUs (Fisher’s exact test *p*-value = 1.0e-4) and between environmental and patient samples (Fisher’s exact test *p*-value = 1.0e-4) (Additional file 1: Fig. S2). Of the 374 ICU admissions involved in the study, 71 were colonized and/or infected with two or more different STs (Fig. S3).

A phylogenetic analysis was performed using Gubbins algorithm [45] on a core genome alignment generated by read-mapping to the PAO1 reference genome. The result showed a very diverse dataset, including isolates from the three major PA phylogroups, which were identified using the reference genomes of PAO1, PA14, and PA7 for phylogroups 1, 2, and 3, respectively [46] (Fig. 1B).

### Genetic diversity

We estimated pairwise genetic distances among strains using three methodologies. 1) Whole genome SNPs (wgSNPs) were obtained using the reference-free Split Kmer Analysis (SKA) toolkit [47]. Split kmers are pairs of sequence kmers that differ by only a single base; consequently, the distance between two sequences is determined by the number of shared split k-mers, corresponding to the number of variable sites. SKA provides rapid and highly accurate analysis of the pairwise core genome (i.e., all sequences shared by a pair of strains), but since different pairs of strains share different fractions of their genome, SKA effectively analyzes the pangenome of the strain collection except for singletons. 2) We also used SKA to identify reference-based core genome SNPs (rbcgSNPs) by using SKA-map to align the split kmers to the reference genomes PAO1, PA14, and PA7. 3) Finally, we identified reference-free core genome SNPs (rfcgSNPs). Using the three phylogroup binning identified with the phylogenetic analysis, we identified, extracted, and concatenated SNPs from aligned single-copy orthologous genes. While this approach has the limitation of lacking paired comparisons between samples from different groups, it still allows for the evaluation of recent transmission (see Methods).

We evaluated the pairwise SNPs difference both within and between STs, and as expected, pairwise differences are smaller within STs than between STs. The number of SNP differences within STs ranged from 0 to 3,570 for rbcgSNPs, 0 to 3,325 for rfcgSNPs, and 0 to 4,577 for wgSNPs. In contrast, the number of SNP differences between different STs ranged from 12 to 44,760 for rbcgSNPs, 5 to 92,076 for rfcgSNPs, and 14 to 45,725 for wgSNPs (Fig. 1C).

The pairwise SNP distances correlate well between the three approaches, although differences were noted for the wgSNP/rbcgSNP comparison using PA7 reference genome. We speculate that this may be due to the greater degree of diversity among phylogroup 3 strains, resulting in poorer read mapping. We also observed a weaker correlation at small pairwise genetic distances for all three phylogroups in the wgSNP/rfcgSNP comparison (Additional file 1: Fig. S4). We speculate that this may be due to greater genetic cohesion driven by similar accessory genomes, which would only be measured in the wgSNP approach. Given the overall high correlation, we elected to proceed primarily using the rbcgSNPs approach with the PAO1 reference genome and the wgSNP approach since it is faster and requires fewer specialized skills to deploy.

### Genetic Candidate Transmission Pairs (gCTPs)

**Fig. 2:**
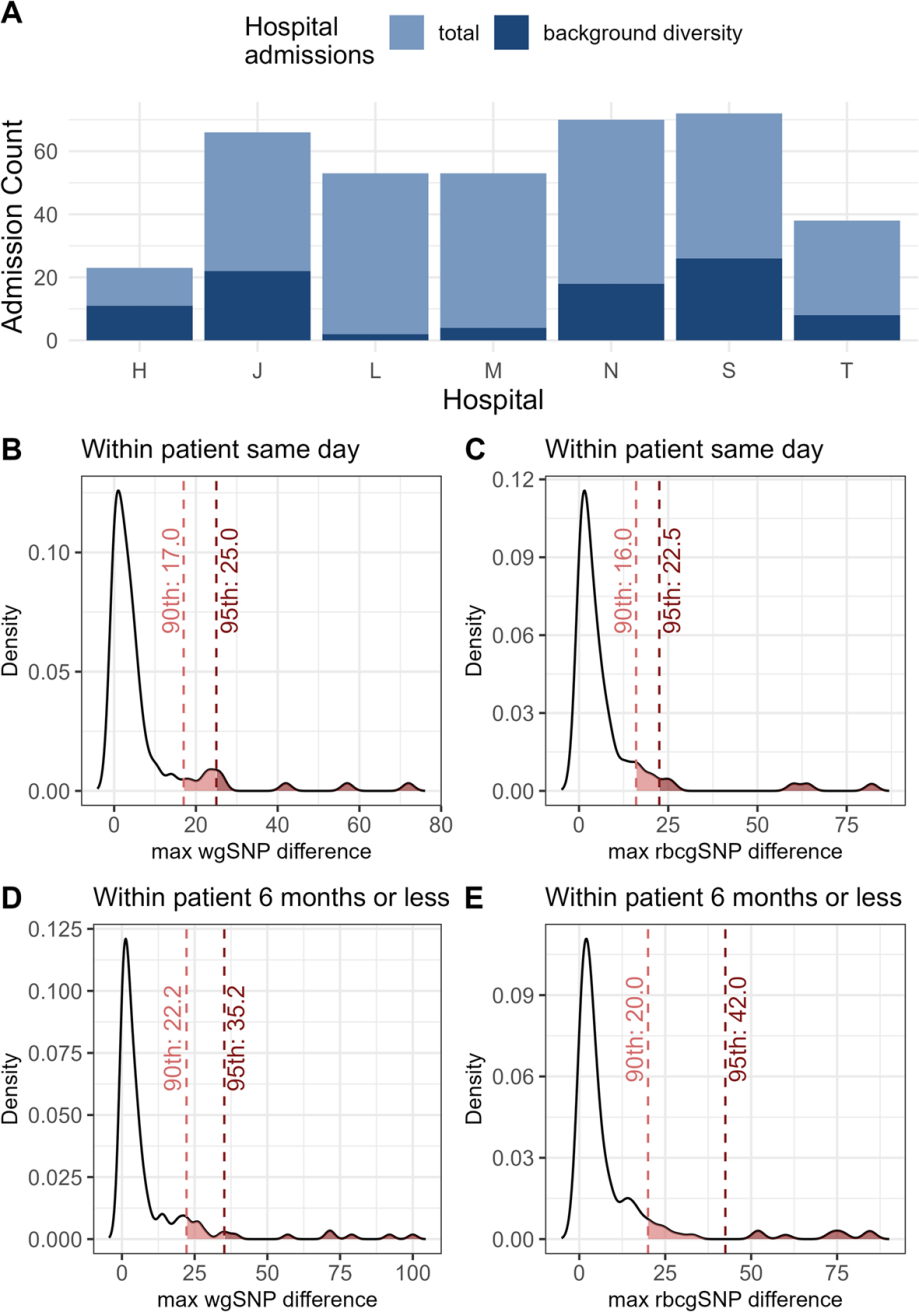
**A** The number of isolates available from ICU admissions for each hospital and the proportion of those that were used to calculate the background diversity. **B** Density plot of the distribution of pairwise whole genome single nucleotide polymorphism (wgSNP) differences between isolates recovered from the same patient on the same day. Vertical dashed red lines mark the 90th percentile (17 SNPs) and the 95th percentile (25 SNPs), indicating the possible values for background diversity. **C** As in panel B for within-patient pairwise reference-based core genome SNP (rbcgSNP) differences. Vertical dashed red lines mark the 90th percentile (16 SNPs) and the 95th percentile (22.5 SNPs). **D** As in panel B for within-patient pairwise wgSNP differences for isolates recovered over a period of six months or less. Vertical dashed red lines indicate the 90th percentile (22.2 SNPs) and the 95th percentile (35.2 SNPs). **E** As in panel B for within-patient pairwise rbcgSNP differences for isolates collected over a period of six months or less. Vertical dashed red lines show the 90th percentile (20 SNPs) and the 95th percentile (42 SNPs)

Our first aim was to identify transmission events occurring between the hospital environment and patients or between different patients using a genetic threshold based on pairwise genetic distance between strains. We identified genetic candidate transmission pairs (gCTPs) based on a threshold of pairwise genetic distance, which establishes the maximum genetic distance expected from the divergence of clonal lineages. Pairwise diversity should be below the threshold within clonal populations and lineages, while mixed populations or highly divergent lineages will fall above the threshold. We used two published methods based on within-host diversity to establish this threshold [48]. First, we estimated the background diversity as the 90th percentile of the maximum pairwise SNP differences between isolates of the same MLST ST, collected from a single patient on the same day [48]. A total of 91 ICU admissions met these requirements, with the number varying significantly depending on the ICU under consideration (Fig. 2A). As expected, the distribution of pairwise SNP differences showed a strong right-skew. We chose to set our genetic distance threshold using the 90th percentile rather than the 95th percentile to limit the impact of outliers (Fig. 2B, C). Using this criterion, the background diversity for the rbcgSNP analysis was 16 SNPs, while for wgSNP analysis it was 17 SNPs. When we eliminated the requirement for same day sampling and included pairwise comparisons from within-patient strains of the same ST isolated up to six months apart, the thresholds for the rbcgSNP and wgSNP rose to 20 and 22 SNPs, respectively (Fig. 2D, E and Table 1). By extending the time between isolates to 6 months, we can approximate a threshold to assess recent transmissions.

**Table 1:** SNP Threshold Calculation using a Linear Mixed Regression Model (LMM)

Method	Reference Genome	BD Same day	BD Six months	Median Intercept	SD Intercept	Median slope	SD slope	LMM threshold
wgSNPs	None	17	22	8.088675	0.168993	0.606548	0.076873	24
rbcgSNPs	PAO1	16	20	6.391023	0.282335	0.29448	0.027426	20
rbcgSNPs	PA14	15	19	6.73176	0.121638	0.55697	0.03271	21
rbcgSNPs	PA7	11	15	6.283655	0.108922	0.415027	0.088397	20

*BD* Background Diversity

The second approach we used to calculate a pairwise SNP difference threshold used a linear mixed regression model (LMM), with SNP distance as the dependent variable and time in months between collection as the independent variable [48]. This approach allowed us to obtain a nucleotide substitution rate which, along with the calculation of background diversity, could be used to estimate a threshold for recent transmissions (defined as six months). We compared two different LMMs: one including both patient admissions and ICU as random effects, and a simpler model that included only patient admissions as a random effect. The explained variance by ICU was negligible, and the Akaike and Bayes information criteria values supported the simpler model choice; therefore, we retained only admission as a random effect. The intercept represents the mean background diversity, and the slope indicates the substitution rate. The samples were de-duplicated, keeping only one per day, and the LMM was fitted 100 times. The final estimates of the intercept and slope were based on the median values of these repetitions. The SNP threshold for recent transmission (recent defined as 6 months) was calculated as: background diversity + (time * substitution rate) * 2, which resulted in a final threshold of 20 SNPs for the core genome and 24 SNPs for the whole genome (Table 1). Table 1 summarizes the genetic thresholds obtained with different methods for core and whole genome approaches. The gCTPs are defined as pairs of isolates with a SNP distance less than or equal to the estimated threshold. Of a total of 2,094,081 pairwise comparisons in the full dataset, 17,537 have a genetic distance below or equal to the wgSNPs threshold of 24, and 2,915 are pairwise comparisons of interest for transmission based on either being environment-patient pairs (1,895) or patient-patient pairs (1,020).

### Epidemiological linkage score: estimating likelihood of transmission between isolate pairs

**Fig. 3:**
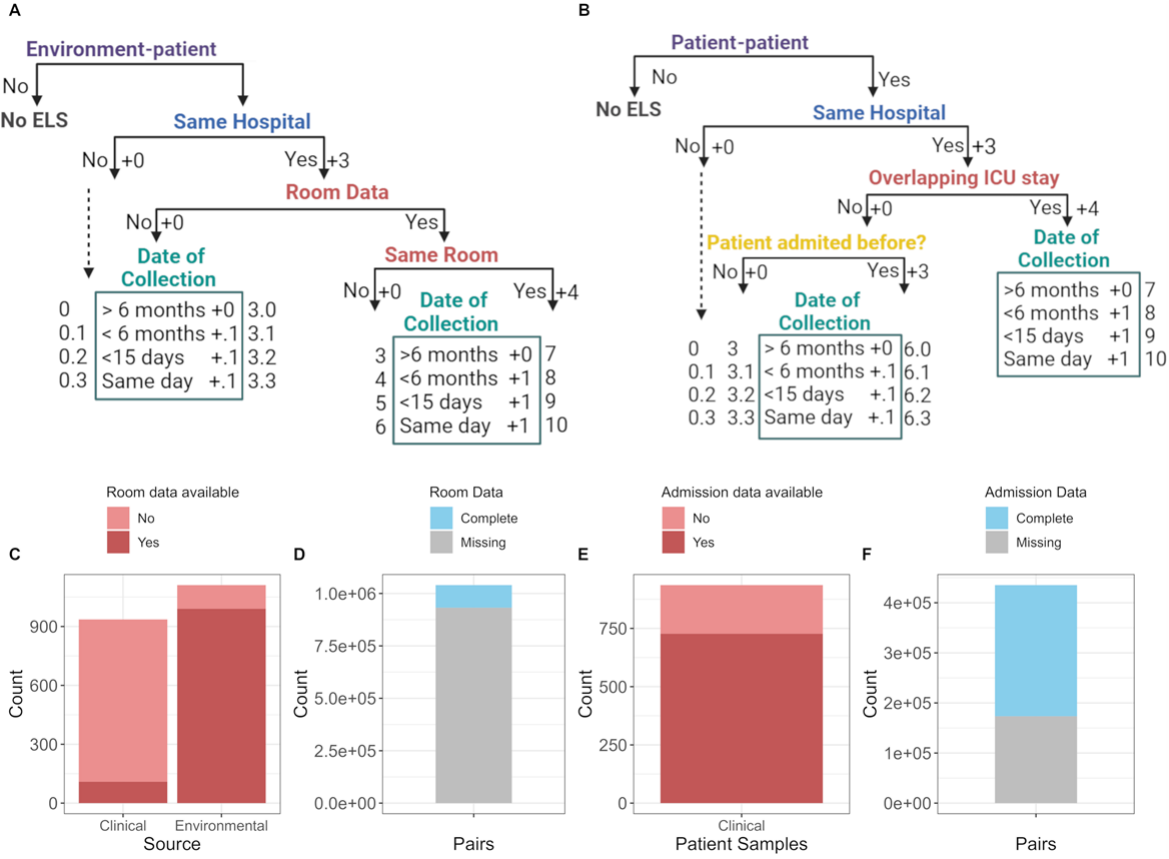
**A** Decision tree outlining the criteria used to calculate the epidemiological linkage score (ELS) for pairs of isolates where one isolate is from a patient and the other is from the ICU environment. If the pair does not meet the environment-patient criterion, ELS = 0.0. If the isolates are from the same hospital, the ELS is incremented by 3 points. Further bifurcation is based on the availability of room data, influencing the score: If room data is available and the isolates are from the same room, another 4 points are added. The date of collection further adjusts the score. **B** Decision tree outlining the criteria used to calculate the ELS for pairs of isolates recovered from different patients. If the pair does not meet the patient-patient criterion, ELS = 0.0. If the isolates are from the same hospital, the ELS is incremented by 3 points. Further bifurcation is based on the availability of admission date to estimate whether the two patients had overlapping stays in the ICU. If the patients had overlapping ICU stays, another 4 points are added. If the pair involve different ICU admissions from the same individual, an additional 3 points are added. The date of collection further adjusts the score even if the stays do not overlap to account for time proximity in the ICU. **C** Availability of room data for patient and environmental isolates. The y-axis represents the count of samples. **D** Availability of full room data for pairs of strains. The y-axis represents the count of pairs. **E** Availability of admission date data for clinical samples. The y-axis represents the count of patient samples. **F** Availability of full admission date data for pairs of strains

Our second aim was to use epidemiological information to identify probable transmission events occurring between the hospital environment and patients or between different patients. No outbreaks were reported during the study; therefore, we had no epidemiologically defined transmission clusters for analysis. As an alternative, we developed an Epidemiological Linkage Score (ELS) calculated from isolate metadata. The ELS score was constructed differently for the different types of isolate pairs (Fig. 3). For environment-patient pairs, the score was based on being in the same ICU, whether the patient had occupied the room the environmental isolate was obtained from, and the time interval between samples. For patient-patient pairs, the score was based on being in the same ICU, having overlapping admissions, and the time interval between the collection of the samples. Unfortunately, the design of the original study meant that most information on the rooms patients occupied over time, and many admission dates for patients were not available. Thus, a high proportion of environment-patient pairs and nearly half of patient-to-patient pairs did not have complete information (Fig. 3D-F).

**Fig. 4:**
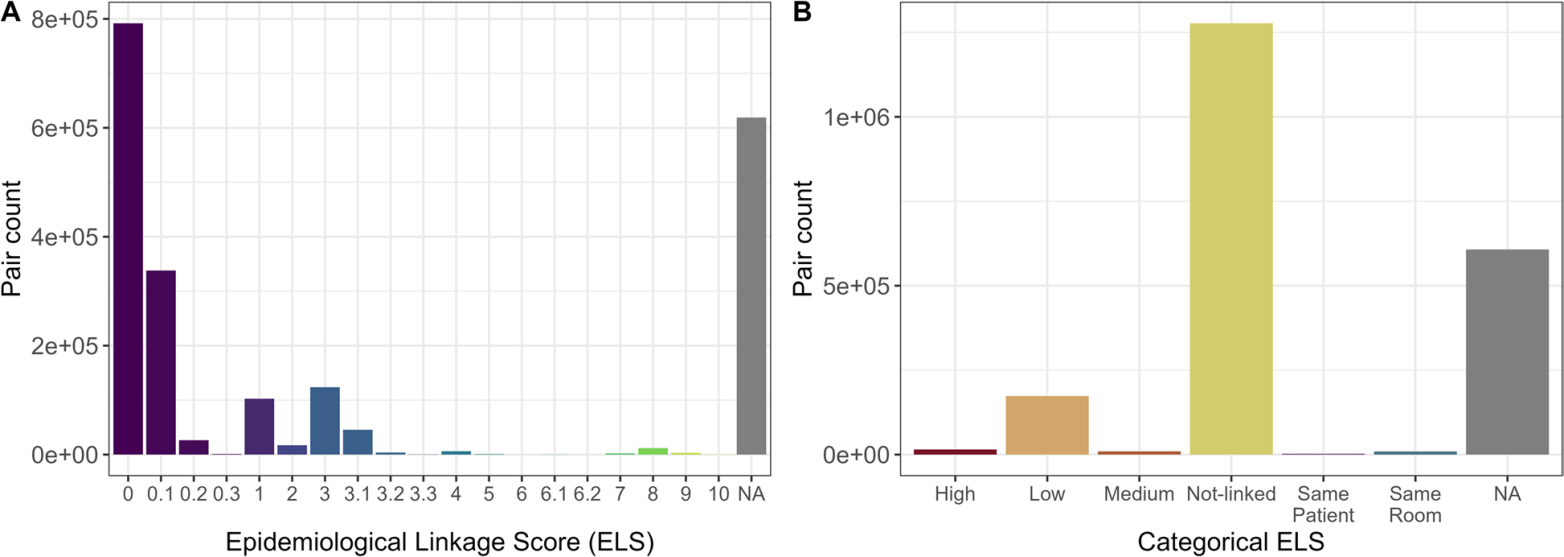
**A** Frequency distribution of ELS for pairs of isolates. The x-axis represents the ELS, ranging from 0 to 10, and the y-axis represents the isolate pair frequency, indicating the proportion of pairs falling within each ELS value. The distribution shows that the majority of pairs have low ELS values. **B** Frequency distribution of categorized ELS scores for the pairs of isolates. The x-axis represents the ELS categorized score labels: High (ELS > = 8), Medium (4 < = ELS < 8), Low (ELS = 3), Not-linked (ELS < 3). NA is assigned to those for pairs of strains that do not have enough data to infer transmission. Pairs of strains isolated from the same patient and room are also shown. The y-axis represents the pair frequency, indicating the proportion of pairs in each category

The ELS ranged from zero to ten (Fig. 4A), and most pairs were either non-transmission pairs (environment-environment and same patient pairs, assigned NA) or were not associated epidemiologically (ELS < 3). Values equal to or greater than eight were considered high epidemiological links that were achieved when environment-patient pairs shared the same room or patient-patient pairs had overlapping ICU stays. ELS values between four and eight were assigned a medium level of epidemiological association, while ELS values equal to three were considered low, and those below three had no epidemiological link. The ELS was discretized for subsequent analysis into high, medium, low, and not-linked categories. For comparison, we also included alongside the categorical ELS score, a category for pairs of environmental isolates from the same room (Same room), and clinical isolates from the same patient (Same patient): these are non-informative for transmission analysis but are obviously epidemiologically linked (Fig. 4B). As previously stated, many isolates were missing some of the information required to calculate the ELS, which influenced the distribution of pairs across each category; more than 70% of pairs assigned to low and not-linked categories had incomplete epidemiological data.

### Epidemiological evidence supporting genetic transmission events

**Fig. 5:**
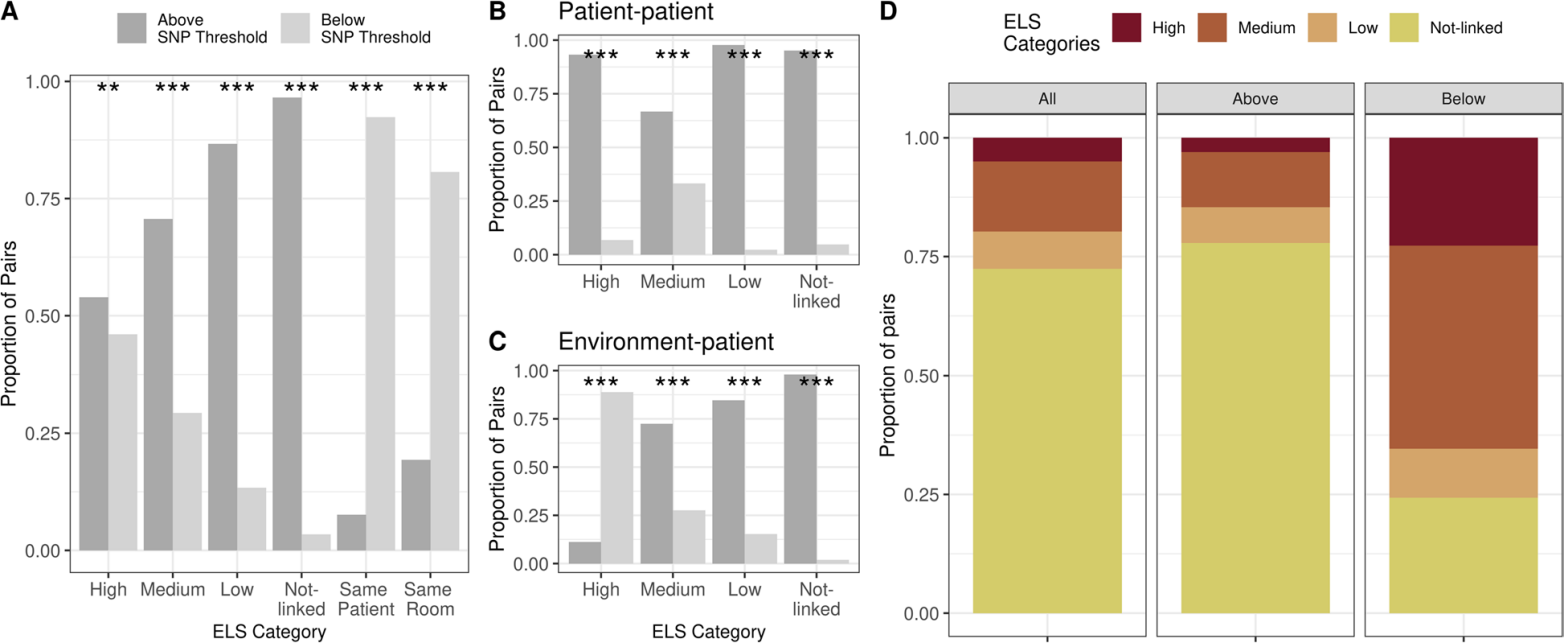
**A** Proportion of pairs of strains above and below the defined wgSNP threshold of 24 for each ELS category including same patient and same room. Significant differences are marked with asterisks, indicating statistical significance. **B** The proportion of patient-patient pairs in each ELS category above and below the SNP threshold. Significant differences are marked with asterisks, indicating statistical significance. **C** Similar to Panel (**B**), showing the proportion of environment-patient pairs in each ELS category, above and below the threshold. Significant differences are marked with asterisks, indicating statistical significance. **D** Comparison of the distribution of the ELS categories when taking into account all pairs, pairs above and pairs below the wgSNP threshold, Only pairs of strains composed of samples from different sources (environment-patient or different patients) with ELS values calculated are shown

A key question is whether genomic sequence data can be rapidly deployed to identify transmission events. To address this question, we stratified the data based on the discrete ELS and compared the distribution of ELS categories between pairs above and below the genetic threshold of 24 SNPs. We restricted the analysis to pairs with complete epidemiological data for the ELS and shared MLST ST, which reduced the number of pairs to 59,352 of which 17,812 are gCTPs (i.e., pairs below the SNP threshold). The gCTPs captured 92% of pairs from the same patient and 81% of environmental pairs taken from the same room, and only 4% of not-linked pairs. Additionally, 88%, 55%, and 80% of pairs with high, medium, and low ELSs, respectively, were above the SNP threshold (Fig. 5A). To explore in more detail, we divided the pairs into patient-patient (Fig. 5B) and environment-patient (Fig. 5C) categories and found that only 4.8% and 1.9% of the non-linked pairs were in the below-threshold category. This is a critical finding since it shows that the SNP threshold very effectively identifies putative transmission events by removing pairs with no epidemiological link (Additional file 1: Fig. S5). We also observed that most of the pairs with a high ELS that fall above the SNP threshold correspond to patient-patient pairs (Fig. 5B). There are several possible explanations for this. One possibility is that clonal divergence may lead to high population diversity within patient reservoirs and transmitted strains. Another is that patient-to-patient transmission is rare, with patients acquiring PA from multiple distinct sources within the ICUs. In this case, overlapping hospital stays (the criteria required for a high or medium ELS) may be too lenient and, therefore, not reflect a strong epidemiological link. To investigate this further, we examined whether patient pairs closer in time were more likely to be genetically related, defined by having a SNP distance below the genetic threshold. We considered pairs “close in time” if the difference between sample collection dates was less than the median of 50 days. A chi-squared test comparing pairs above and below the SNP threshold yielded a *p*-value of 0.015, suggesting a significant association between temporal proximity and genetic relatedness. This implies that patient-to-patient transmission may indeed be occurring.

Next, we stratified the data based on the pairwise genetic distance into three groups: all pairs, pairs below the SNP threshold, and pairs above the SNP threshold. We then calculated the proportion of each ELS category within each group (Fig. 5D). Not surprisingly, most pairs above the genetic threshold are not linked, while the majority of gCTPs (i.e., pairs below the genetic threshold) have some epidemiological link. There is a significant increase in ELS high, medium, and low pairs in the gCTP below-threshold group compared to the above-threshold group and a decrease in not-linked pairs (Fisher’s Exact Test for Count Data: medium, *p*-value = 4.58e-05, low, *p*-value = 2.76e-16; Not-linked, *p*-value < 2.2e-16). This suggests that the estimated threshold of 24 SNPs allows us to identify pairs of isolates that are potentially related by transmission, of which a significant portion also have epidemiological support.

### Transmission clusters reveal persistent strains in the ICU environment

**Fig. 6:**
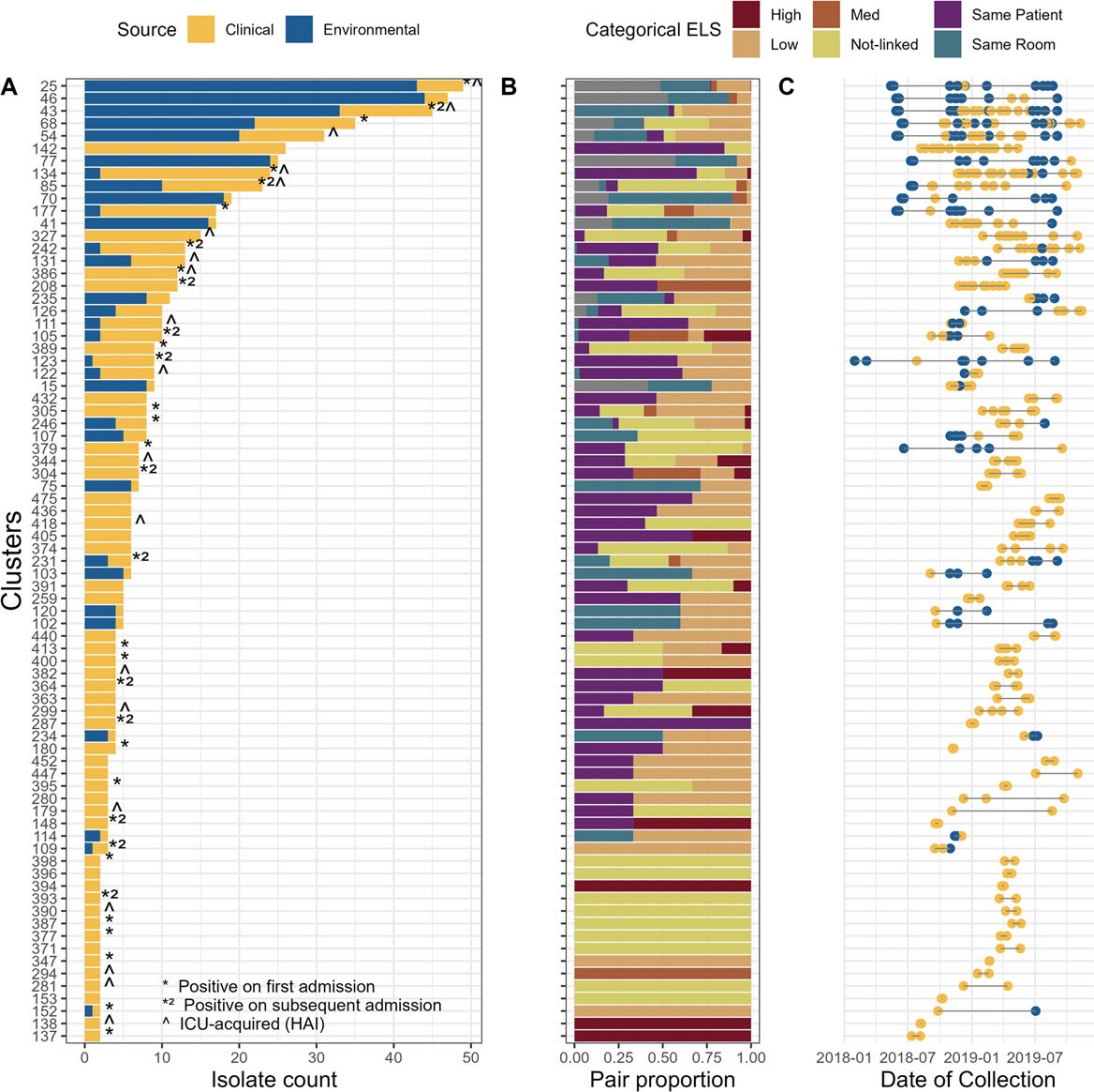
**A** The number of isolates in each transmission cluster, showing the source of isolation proportion. Transmission clusters were defined using average linkage clustering with a threshold of 24 SNPs. The y-axis lists each transmission cluster, ordered by cluster size. The x-axis is the number of isolates assigned to the cluster. **B** The proportion of pairs of strains within each transmission cluster binned by ELS categories. The y-axis represents the transmission clusters as in panel (**A**). **C** Collection dates for each isolate in each transmission cluster. The y-axis represents the transmission clusters as in panel (**A**), and the x-axis the collection dates

Finally, we sought to identify possible transmission clusters that can link multiple strains and transmission events beyond the pairwise linkages found above. Clusters were generated using average linkage clustering and the wgSNPs threshold of 24. A total of 276 clusters involving 1822 isolates were identified (Additional file 1: Fig. S6). Only 224 isolates were not assigned to any cluster. Clusters can be composed exclusively of environmental isolates (95 clusters), exclusively of patient (i.e., clinical) isolates (150 clusters), or a combination of both (31 clusters). Clusters that include patient samples may include isolates from multiple patients or multiple admissions from the same patient (Additional file 1: Fig. S7). To study transmission events, we selected only those clusters with different patients or environment-patient pairs. A total of 77 clusters met this criterion, with 46 including at least one patient-patient gCTP and 31 including at least one environment-patient gCTP (Fig. 6A). Surprisingly, 38 transmission clusters involved more than one ICU (Additional file 1: Fig. S8), pointing to a non-trivial amount of inter-hospital transmission or common sources that result in exposures to the same strain in different ICUs [49]. We also found that 19 of the 77 transmission clusters found contain samples previously identified as HAI.

Transmission clusters exhibited variable time ranges. While some clusters identify groups of isolates collected only a few days apart, others include isolates recovered over nearly two years of sampling (Fig. 6C). A closer inspection of the gCTPs shows that a large proportion of the links that extend over long time periods are environmental isolates from the same ICU room. This suggests that there are persistent environmental reservoirs of isolates that are sources of contamination in the ICU (Additional file 1: Fig. S9). Environmental sampling conducted prior to patient sampling revealed that 15 transmission clusters contained environmental isolates associated with patients that were collected earlier in time, suggesting possible acquisition of *PA* from the hospital environment (Fig. 6C). Of these, eight clusters include isolates from previously identified ICU-HAIs.

A total of 716 isolates are part of the 77 transmission clusters (35% of the samples under study), encompassing 409 patient isolates associated with 154 distinct patients (43% of all patients). There were 59 (16%) patients with links to environmental samples while 95 patients (26%) were found to be involved in probable patient-patient transmission. Transmission clusters commonly included pairs of strains with different ELSs and pairs with missing epidemiological metadata (i.e., room of isolation) (Fig. 6B). The impact of missing data, specifically the lack of information on the room occupied by a patient at a given time, primarily affects the ‘low’ and ‘not-linked’ categories, which show a high percentage of pairs with incomplete information (93% and 85% pairs correspondingly with missing data). This leads to an overestimation of low levels of epidemiological association, particularly between patient and environmental samples, since high epidemiological links between patients requires overlapping hospitalization periods.

**Fig. 7:**
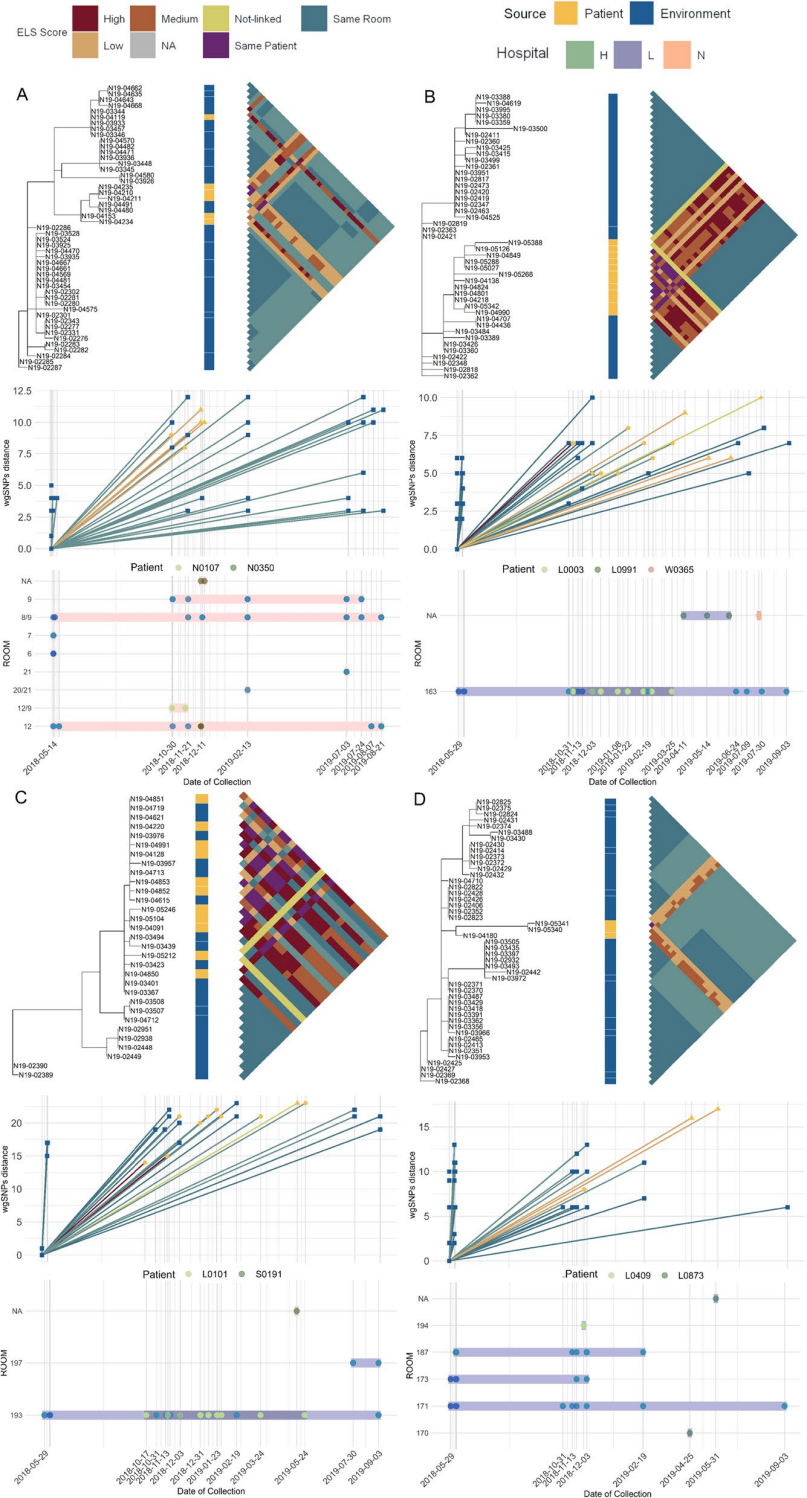
Temporal and spatial distribution of isolate sources in transmission clusters. The figure is based on a non-clonal subset of each cluster. **A** Cluster 54, **B** Cluster 25, **C** Cluster 43, **D** Cluster 46. Each panel consists of three subpanels. Top: neighbor-joining tree based on wgSNPs distances and rooted using the focal sample (earliest sample in the cluster). The source of isolation (i.e., patient or environmental) for each isolate on the tree is presented in the metadata column to the right. The triangular matrix is a pairwise heatmap of the ELS category for each pair of isolates. The ELS category for each pair is the point of the triangle connecting the two strains. Middle: The date of isolation (x-axis) by wgSNPs distance between the focal isolate and other isolates in the cluster (y-axis). The focal isolate is defined as the earliest isolate recovered from that cluster. Lines between isolates are color-coded by that pairs ELS category. Bottom: date of isolation by room of isolation. Each is an isolate color-coded by the source, i.e., environmental or individually identified patients. The segments behind the dots are color-coded based on the hospital ICU of isolation and extend for the temporal range of isolates in that particular room

Using the estimated SNP threshold and the average clustering method, we were able to identify clusters that confirm transmission cases previously identified in a study conducted by our group on a subset of the data using pre-defined epidemiological and genomic criteria [15]. In this prior study, environment-patient transmission (referred to as sink-to-patient transmission in [15]) was defined as PA isolation from patients not PA-colonized at admission, with genetic distance of less than 20 SNPs between patient and environmental isolates collected from a room they occupied between 3 to 14 days prior to the first patient isolate. Three of these cases are shown in Fig. 7A-C. The present analysis identified these three events and was able to link the patient isolates with additional samples that were obtained from follow-up collections in the rooms where transmission was detected. These results indicate that the environmental source of contamination can persist for well over a year and be responsible for the infection of multiple patients. The transmission clusters were also able to link isolates that came from multiple ICUs, e.g., both patient S0191 from ICU:H (Fig. 7A) and patient W0365 from ICU:T (Fig. 7C) clustered with environmental and patient samples from ICU:L. We also identified previously unidentified transmission clusters; one example is shown in Fig. 7D.

## Discussion

In this study, we evaluated the transmission dynamics of 2046 fully sequenced *P. aeruginosa* (PA) isolates collected from the Intensive Care Units (ICUs) of seven hospitals in the Greater Toronto Area (Ontario, Canada) over a two-year period. The sampling was designed to assess the importance of environmental reservoirs as sources of nosocomial infections, and to determine if genomic data could be used to rapidly and easily identify ICU-associated transmission events.

Sinks, faucets, drains, humidifiers, and respiratory equipment have been identified as environmental reservoirs of PA in the hospital environment [14, 16, 50–54]. Despite having established the importance of environment-to-patient and patient-to-patient transmission, we still have only a limited understanding of the relative likelihood and importance of transmission from different environmental sources, the amount of genetic diversity segregating in environmental reservoirs, and the amount of genetic diversity that can be expected in clonal lineages dispersed between reservoirs and patient populations. As is always the case with an evolving research area, pursuing and interpreting these questions are made more difficult by the lack of standardized approaches and analyses.

Genomic approaches provide objective, high-resolution data that can be used to supplement existing epidemiological methods. Unfortunately, analyzing and interpreting genomic data typically relies on the specialized and sophisticated genome analysis tools and approaches that are usually the domain of skilled bioinformaticians. We sought to determine if this complex workflow could be largely replaced using the Split-Kmer Analysis (SKA) Toolkit that was developed specifically for bacterial genomic epidemiological studies [47]. This approach is fast, reproducible, and relatively easy to employ and interpret, even by individuals with limited bioinformatic skills. Importantly, the wgSNP SKA approach does not require a reference genome, and in effect, it performs a pangenome diversity analysis, unlike reference-based approaches that are limited to the core genome. SKA has already been successfully used for epidemiological surveillance investigations and for the analysis of recent transmission events [55–57], although this study is unique in its use of the epidemiological linkage (ELS) to generate epidemiological candidate transmission pairs (eCTPs) and evaluate genomic candidate transmission pairs (gCTPs), as well as the generation of transmission clusters to identify larger transmission chains. The one downside to SKA is that its results are not entirely portable, in the sense that an existing SKA-based SNP distance analysis cannot be supplemented with new strains unless all genome sequences are available for analysis. Nevertheless, this issue will not impact work being done within a laboratory and is ameliorated somewhat for others by the requirement of data submission to genomic data repositories such as NCBI and EMBL.

Even when genomic epidemiological data is available, it is still challenging to determine if any two isolates are clonemates. There is no universally agreed upon SNP threshold for defining recently diverged clonal lineages, much less a universally agreed upon method to determine this value. We addressed this by employing two independent methods. The first method was based on the empirical distribution of pairwise SNP differences between strains isolated from the same patient over a maximum of six-months. The second method employed a linear mixed model (LMM) using SNP distance as the dependent variable and admission as a random effect to predict time (in months) between collection as the independent variable. These two methods produced highly consistent results with thresholds of 22 and 24 SNPs using wgSNP for the empirical and LMM approaches, respectively, and 20 SNPs using rbcgSNPs with both methods.

SNP thresholds for studying PA outbreaks and transmission have been previously calculated and align closely with our estimates. As discussed in the introduction, prior work from our group used a subset of the data presented here and a different core genome analysis to estimate a threshold of 20 SNPs [15]. Weimann and colleagues [5] identified a threshold of 26 SNPs for transmission clusters using a reference-based approach and the 95th percentile of within-patient diversity in 81 patients. A study of a PA ST-621 outbreak from a U.S. military hospital found that the average pairwise distance between strains was 38 SNPs, while the isolates from two phylogenetically defined subclones had an average distance of 24 and 31 SNPs [43]. A similar finding of a 25 SNP threshold was recently reported in a retrospective study of 14 PA outbreaks at the Lausanne University Hospital (Switzerland) over a 15-year period [42]. The authors of this same paper also reviewed 19 published PA outbreak studies and found that the thresholds ranged from 15–25 SNPs [42].

Sink drains were identified as an important environmental source of PA, with long-term persistent isolates in the ICU environment contributing to patient infections. Environmental reservoirs were recently identified as a major source of nosocomial PA infections in another study of a prolonged outbreak in a single institution, with isolates differing by zero to four SNPs despite being collected even three years apart [43]. A similar scenario was recently reported in a retrospective study of 14 PA outbreaks at the Lausanne University Hospital (Switzerland) over a 15-year period [42]. The authors found that environmental isolates had lower genetic diversity and showed slower genomic evolution compared to clinical isolates, possibly due to reduced selective pressure in the absence of antibiotic treatment. The relevance of microbial contamination from the built environment in the ICUs is a well-known problem [51], though the relative contribution of different sources is still not clear and can vary for different bacterial species [54]. Patient-to-patient transmission via the hands of staff has also been identified as an important source [25]. We identified possible patient-to-patient transmission involving 26% of the patients included in the study; this may represent inadequate hand hygiene practices. However, we also identified patients and sinks with highly related strains in different intensive care units, emphasizing the significant gaps in our understanding of the reservoirs and mechanisms of transmission of PA in ICUs. After defining a genetic threshold for transmission, we asked if this value was supported by more traditional epidemiological methods. While we confirmed that most epidemiologically defined transmission events (i.e., eCTPs) were also found to be genetically linked (i.e., gCTPs), we also found many gCTPs with no epidemiological link. A closer examination of these strain pairs showed that the lack of agreement was largely due to the lack of strain metadata. This was particularly problematic with respect to patient room data, largely because room data was typically recorded only when an ICU-associated healthcare-associated infection (ICU-HAI) was detected or suspected. Importantly, the lack of metadata resulted in these pairs being categorized in the ‘low’ and ‘not-linked’ ELS categories (Fig. 3), effectively artificially inflating the number of false negatives.

Despite the metadata limitation, we did have enough strains with full data to confirm that a strong epidemiological linkage was associated with closely related strains, i.e., 48.7% of patient-patient pairs below the 24 SNP threshold (gCTP) showed at least some linkage (eCTP), while 70.4% environment-patient gCTPs were also eCTPs. Similarly, 83.8% and 88.6% of patient-patient and environment-patient pairs what exceed the SNP threshold (were not gCTP) were also epidemiologically unlinked (not dCTPs). This approach supports a scalable model for integrating genomic and epidemiological data. Future work may benefit from more comprehensive metadata.

We found that 26% of all patients carried strains that were linked to at least one putative patient-to-patient transmission. These findings support the importance of evaluating infection control strategies, particularly with respect to environmental reservoirs associated with hospital water supplies. However, we also identified very closely related strains from patients and sinks in different ICUs, emphasizing the significant gaps in our understanding of the reservoirs and mechanisms of transmission of PA in ICUs.

This study approach supports a scalable model for integrating genomic and epidemiological data. Nevertheless, further Future validation of genomic data as a supplement or even replacement for traditional epidemiological data will require work may benefit from the a more comprehensive and regimented collection of metadata. Ultimately, we believe that the success of SKA using wgSNPs in identifying epidemiological links and clusters, along with its ease of use and speed offers significant advantages over other, more specialized methods.

## Conclusions

This study reports a rigorously determined genetic distance threshold that enables rapid and reliable identification of *P. aeruginosa* transmission events in hospitals. The threshold is further supported by independent studies reporting similar values. It demonstrates that the SKA toolkit provides a powerful method for identifying transmission events even when there is no information beyond the source of isolation. The SKA method is also relatively straightforward and accessible, requiring minimal bioinformatic expertise. We were able to validate the approach by pairing it with our custom epidemiological linkage score (ELS), and then extend the analysis by performing clustering on the genetic data to identify transmission clusters. This study highlights the role of contaminated sink drains as significant reservoirs for PA transmission in ICUs and provides evidence for patient-to-patient transmission. The findings underline the need for effective infection control measures to minimize the risk to patients posed by contaminated sinks and other environmental sources. The ability to rapidly and accurately identify transmission events using this approach will improve our ability to rapidly identify transmission events and implement infection control practices that will ultimately improve patient outcomes in healthcare settings.

## Methods

### Sample collection

The samples used in this study were collected prospectively as part of a randomized controlled trial of standard versus copper sink drains conducted in seven ICUs in the Great Toronto Area, Ontario, Canada, from 2017 to 2019. *P. aeruginosa* (PA) isolates collected included all clinical isolates from patients in participating ICUs, isolates from the screening of surveillance rectal swabs to identify colonization with PA, and environmental isolates from ICU sink surfaces, basins, and drain tailpieces, air proximal to sinks, and faucets. Clinical isolates were collected continuously from July 2018 to December 2019, while the environmental samples were collected on seven occasions between December 2017 and August 2019. Isolation source, patient code, ICU, and collection date are available for all isolates, while more detailed information, such as room and admission date, are only available for a subset of the sample. Ethics approval with a waiver of consent was granted by review boards at each participating hospital. Details on microbiological methods and DNA extraction can be found in [15].

### DNA sequencing and genome analysis

Whole genome sequencing (WGS) was conducted on isolates from patients with ICU-acquired PA, and sink (drain/air/faucet) isolate(s) from rooms/bedspaces, using the Illumina NextSeq 500/550 at the National Microbiology Laboratory in Winnipeg, Canada. Sequencing data is available in PRJNA1207498 (SUB14981786, SUB14994327, SUB14996233). Raw read data was trimmed using Trimmomatic [58] (LEADING:3 TRAILING:3 SLIDINGWINDOW:4:15 MINLEN:80) and assembled with Spades v3.14.1 (—careful -k 21,33,55,77,83,91,101,113,121,127 –mismatch-correction) [59]. Annotation was performed with Prokka [60]. Reference genomes were obtained from RefSeq database (PAO1:GCF_000006765.1, PA14: GCF_000404265.1, PA7: GCF_000017205.1) and re-annotated with Prokka. Genome assembly quality was also assessed with CheckM v1.1.3 [61] and assemblies with completeness below 99%, contamination above 1%, and strain heterogeneity above 0 were removed from further analysis.

### Phylogenetic reconstruction

A pangenome was obtained using PIRATE [62] with 90 and 95% amino acid identity thresholds and including three reference genomes, PAO1, PA14, and PA7. The pangenome consisted of 29,570 gene families, of which 5,091 were classified as core. The core genome alignment was obtained using the supporting perl script provided by PIRATE, create_pangenome_alignment.pl, with dosage 1 to include only single-copy genes and a pre-filtered list of gene families present in 100% of the samples (strict core). A SNP alignment was created using snp-sites [63] and a phylogeny constructed with RaxML [64] using parameters -m AST_GTRGAMMA -asc-corr = lewis.Visualization was performed with ggtree [65] in R.

### Genetic distance estimation

Whole genome pairwise genetic distances were calculated using SKA. Split kmer files were created from genome alignments using the fasta sub command with the default kmer size of 15. Pairwise SNP distances between all isolates were then calculated using ska distance with default parameter settings. SKA was also used to obtain reference-based core genome SNP distances. Split kmers were mapped to three reference genomes, PAO1, PA14 and PA7 using ska sub command align and pairwise SNP differences obtained using ska distance. Reference-free core genome SNPs were identified separately for the three major PA phylogroups. For each phylogroup, pangenome analysis was conducted using PIRATE with 97% and 98% amino acid identity thresholds, incorporating the respective reference genomes PAO1, PA14, and PA7. Core genes, defined as single-copy genes without putative fission events and present in all isolates and their reference genome, were identified using an in-house R script based on the PIRATE ordered gene family output. A total of 2,988 out of 33,505 genes, 3,789 out of 23,016 genes, and 2,389 out of 11,027 genes were identified as core genes for PAO1, PA14, and PA7 phylogroups, respectively. Core gene sequences for isolates, excluding the reference genome, were then extracted, concatenated into core alignment using an in-house Python script, and SNPs were identified via *snp-dist.* Pearson correlations between SNP distributions, estimated using different methods, were calculated using the cor() function in R.

### Genetic threshold

SNP thresholds were calculated using two different methods based on within host genomic diversity. This analysis was restricted to isolates from patients with admission data. We first calculated SNP and time distances between all pairs of isolates from the same patient admission, if a patient was admitted more than once, each admission was processed independently. We then excluded pairwise comparisons involving isolates from different STs to exclude mixed infection comparisons. We also removed outliers defined as pairwise comparisons with SNP distances above 100 because recent transmission occurring at a greater genetic distance is extremely unlikely.

The first approach consists of estimating the background diversity or pre-existing diversity [48], which corresponds to the maximum SNP distance between samples obtained from the same patient on the same day. Usually, background diversity corresponds to the 95th percentile of this distribution but we used 90th percentiles since the distribution was very right skewed being highly influenced by outliers. In order to get a SNP threshold to identify recent transmission, the maximum SNP distance analysis within patients was extended to pairwise comparisons between samples taken for the same patient up to 6 months apart and the 90th percentile of that distribution used as threshold.

The linear mixed model (LMM) approach from [48] consists of fitting a LMM using SNP distance as the dependent variable and time between samples as the independent variable. In this model the intercept can be interpreted as the pre-existing diversity and it is allowed to vary by patient admission (random intercept). The reported slopes and intercepts are the median values resulting from sub-sampling and running the LMM 100 times as suggested in [48] since the number of samples per patients is different. The median slope is interpreted as the substitution rate and used to estimate a SNP threshold for recent transmission analysis with the following formula: SNP cutoff = (90th percentile of the background diversity at time zero) + (substitution rate over 6 months × 2). The model was fit using *lmer* function in the R package *lme4* [66].

### Epidemiological score

The epidemiological link score (ELS) was designed to assess how likely it is that the isolates in the pair are epidemiologically linked, and therefore how likely they are to be part of a cross-transmission event. The epidemiological data available for all samples was collection date and ICU. Room data was available for 990 out of 1111 environmental samples and 109 out of 936 patient samples. Admission date was available for 727 of 936 patient samples.

The ELS was calculated for pairs of isolates from different sources, either patient-environment pairs or pairs between different pairs (i.e., patient-patient pairs), using slightly different criteria for each.

For patient-environment pairs, the score for any pair starts at zero. If the samples correspond to the same ICU, 3 points are added; otherwise, it remains at zero. Then, it is evaluated whether both samples have room information. If they do not, the temporal distance between them is assessed, adding 0.1 if they are separated by less than 6 months, less than 15 days, or if they were obtained on the same day. If room information is available for both samples, it is assessed whether they were obtained from the same room, which adds 4 points; if not, it adds zero. Then, the temporal distance between the samples is evaluated: if they are separated by more than 6 months, no points are added to the score; if they are separated by less than 6 months, 1 point is added, less than 15 days adds 1 point, and if they were obtained on the same day, 1 point is added. The score is cumulative, so the maximum that can be obtained is 10 (the minimum is zero).

For patient-patient pairs, the score starts at zero. If the samples correspond to the same ICU, 3 points are added; otherwise, it remains at zero. Then, it is evaluated whether their ICU stays overlap (instead of room location due to the movement of staff attending to these patients between different rooms in the ICUs). If we find overlap, 4 points are added to the score, if no overlap is detected we evaluate whether the patient has previous admission, if they do, 3 points are added to the score. Time distance is evaluated as before, adding 1 for overlapping ICU stay pairs and 0.1 otherwise. The numeric score was categorized into low, medium and high epidemiological links according to the following criteria: pairs with ELS from zero to 3 were assigned low, between 4 and 7 were assigned medium and 8 and above pairs were considered to have a high epidemiological link. Pairs of isolates from the same patient were labeled as *Same patient* and pair of isolates from the same ICU room where labeled as *Same room*. All other possible pairs were assigned NA for the ELS.

### SNP cutoff evaluation and transmission clusters

The proportion of each ELS category above and below the genetic threshold was tested for significance using Fisher’s Exact Test for Count Data with simulated *p*-value (2000 replicates) in R. Clusters were generated using the wgSNP distances. Average linkage clustering with a SNP threshold of 24 SNPs was performed with hclust function in R, with method = ‘average’. Transmission clusters were defined as clusters containing at least one environmental and one patient sample or samples from different patients. We utilized Python and the NetworkX library [67] to construct undirected networks where nodes represented individual isolates and edges connected isolates within the same cluster based on genomic similarity. Visualization was performed with Cytoscape [68].

## Supplementary Information

Additional file 1. Contains all supplemental figures and figure legends.

## Data Availability

Raw sequence data is available under BioProject PRJNA1207498 [69]. Analysis code is available under an MIT License at https://github.com/luciagrami/PA_snp_threshold and https://doi.org/10.5281/zenodo.18259627 [70]. Datasets are hosted on Zenodo under the following DOI: https://doi.org/10.5281/zenodo.18259627 [71]. Graña-Miraglia L, Hu X, Volling C, Mataseje L, Mulvey MR, McGeer A, Guttman DS: Epidemiological studies of Pseudomonas aeruginosa in ICUs. BioProject PRJNA1207498. National Center for Biotechnology Information. https://www.ncbi.nlm.nih.gov/bioproject/?term=PRJNA1207498 [69]. Graña-Miraglia L, Guttman DS: PA_snp_threshold. Zenodo. https://doi.org/10.5281/zenodo.18259627 [70]. Graña-Miraglia L, Hu X, Volling C, Mataseje L, Mulvey MR, McGeer A, Guttman DS: Genomic and epidemiological identification of Pseudomonas aeruginosa transmission chains and in hospital ICUs. Zenodo. https://doi.org/10.5281/zenodo.18259627 [71].
