# Supplementary material for: Genomic and epidemiological identification of Pseudomonas aeruginosa transmission chains and in hospital ICUs
Source: Genome Biol. 2026 Feb 20;27:107. doi: 10.1186/s13059-026-04005-1 (PMC13032602; doi:10.1186/s13059-026-04005-1)

**Fig. S1. Overview of admission swab results and clinical outcomes in ICU patients.**
**(A)** Sunburst plot showing the distribution of all ICU admissions according to admission swab status and clinical positivity. The inner circle represents all admissions. The middle ring shows admission swab results (positive or negative), and the outer ring further classifies cases by clinical swab status (clinical positive, clinical negative, or no clinical swab). Numbers indicate counts, and percentages are relative to the total number of admissions. **(B)** Sunburst plot summarizing outcomes among ICU patients with healthcare-associated infections (HCI). The inner circle shows overall outcomes (survived vs. died with infection contributing). The outer ring details infection types associated with each outcome, including ventilator-associated pneumonia (VAP), pneumonia, bloodstream infection (CLABSI), surgical site infection (SSI), urinary tract infection (UTI), catheter-associated UTI (CAUTI), and SSTI. Numbers indicate counts, and percentages are relative to the total number of ICU HCI cases.


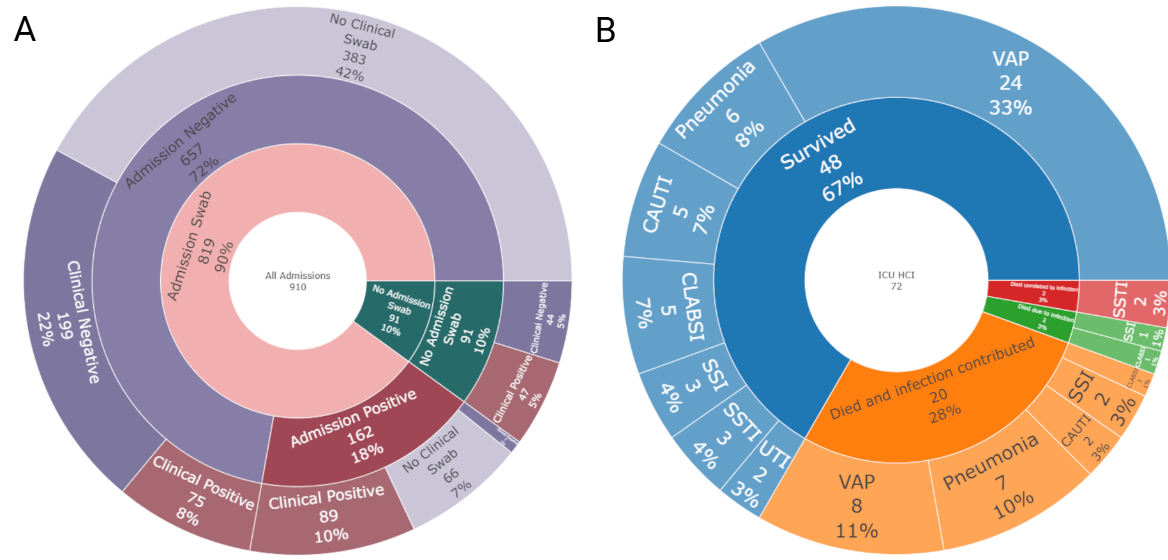


**Fig. S2. Distribution of sequence types by source.**
Polar bar plot showing the number of isolates per sequence type (ST), stratified by source. Bars represent counts of isolates recovered from the environment (red) and from patients (blue) for each ST, arranged around the circle. The radial axis indicates the number of samples. Sequence types are labeled along the circumference, including common STs and categories grouping rare types (e.g., “Other”) or previously unreported types (“Novel”). The accompanying table summarizes the exact counts of environmental and patient isolates for each ST.


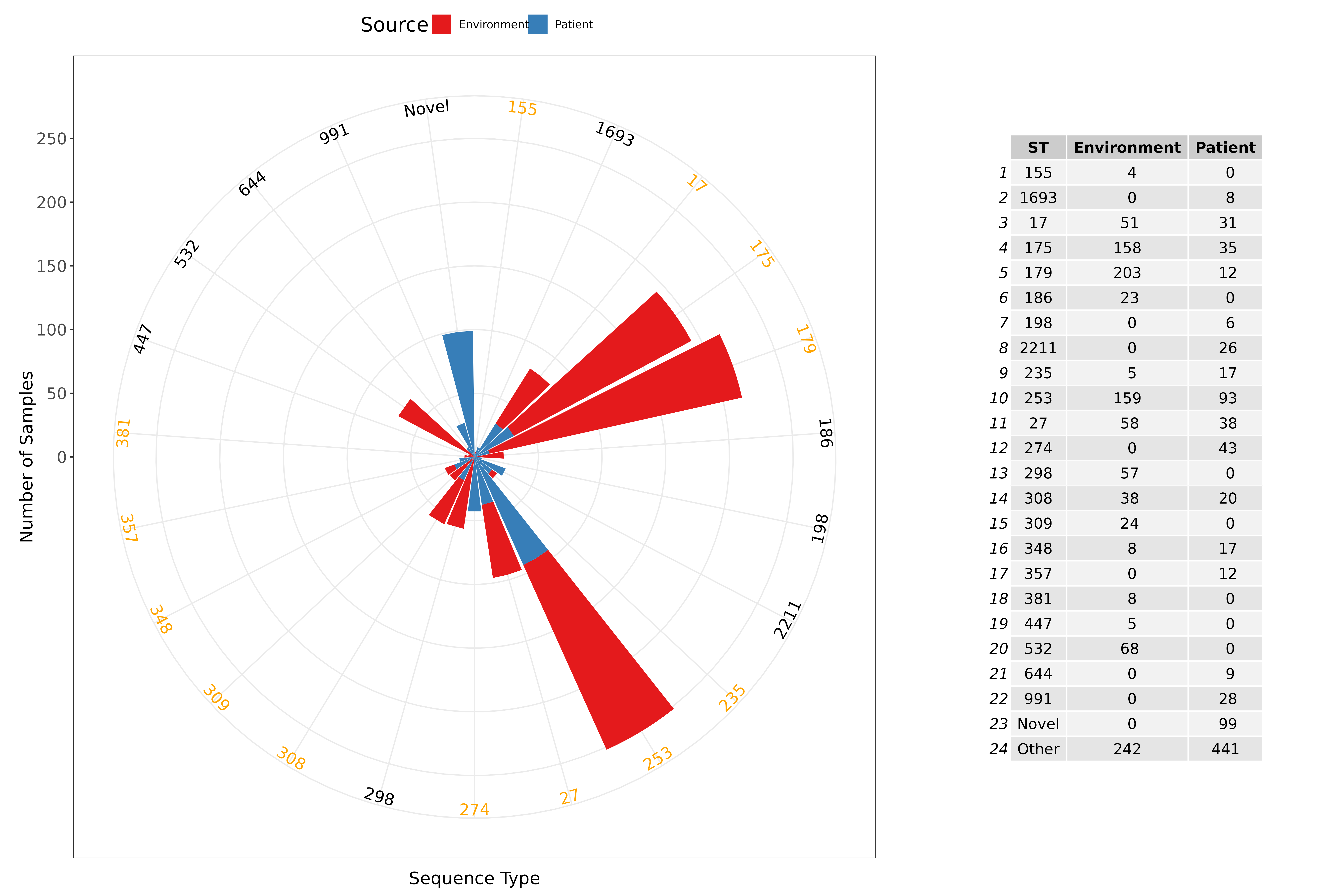


**Fig. S3. Whole-genome distance between isolates by patient and ICU.**
Scatter–boxplot visualization of pairwise whole-genome distances between isolates, grouped by patient and stratified by ICU (H, J, L, M, N, S, and T). The x-axis shows individual patients, and the y-axis indicates whole-genome distance. Each point represents a pairwise comparison between isolates from the same patient. Points are colored according to sequence type (ST) concordance, with pairs sharing the same ST shown in pink and pairs with different STs shown in blue.


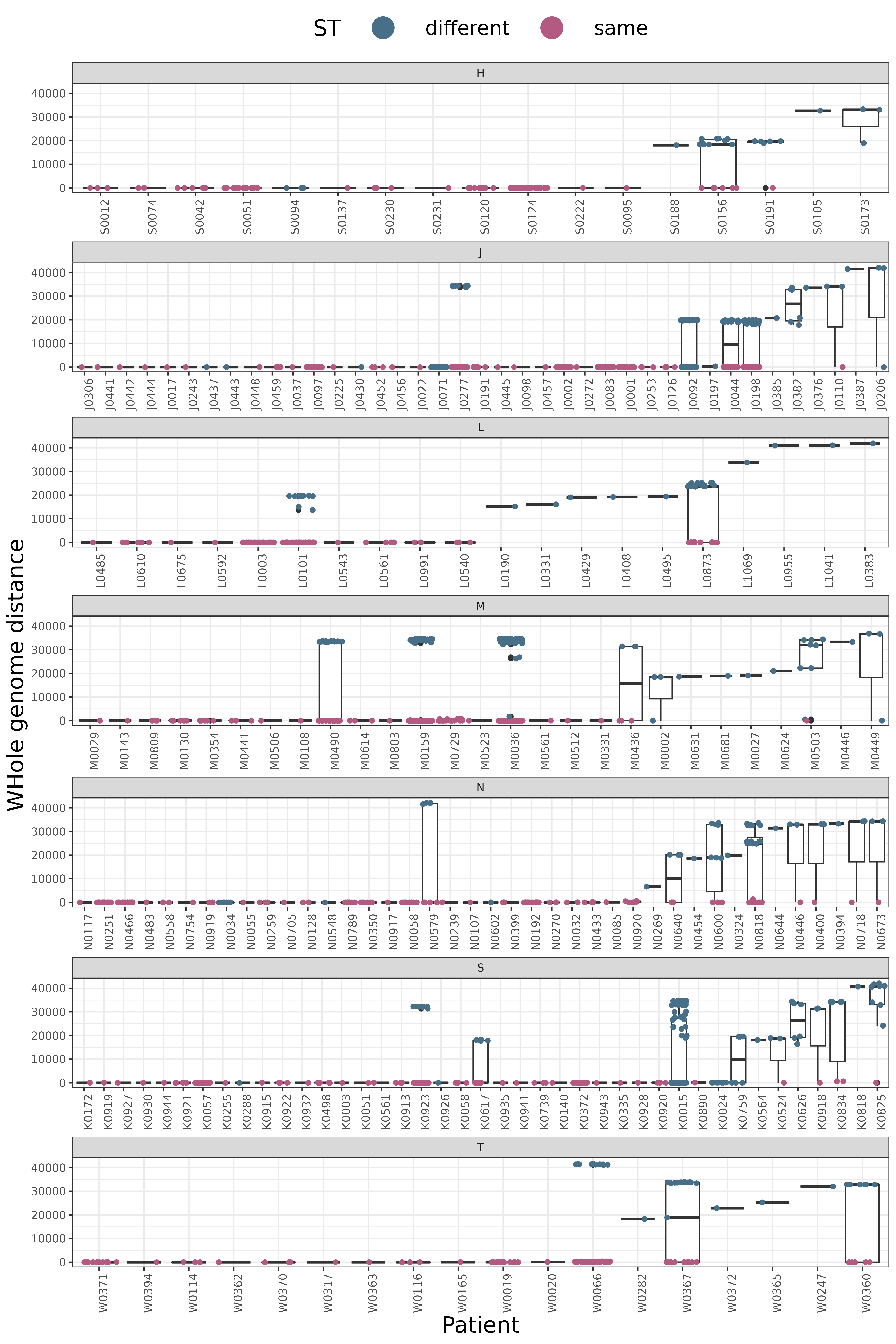


**Fig. S4. Relationship between whole-genome and core-genome pairwise distances.**
Scatter plots showing the relationship between pairwise whole-genome SNP distances (wgSNPs) and core-genome SNP distances for three reference strains (PAO1, PA14, and PA7). **(A)** Comparison between whole-genome SNP distances and reference-based core-genome SNP distances (rbcgSNPs). **(B)** Comparison between whole-genome SNP distances and reference-free core-genome SNP distances (rfcgSNPs). For each reference strain, the top row includes all isolate pairs, while the bottom row is restricted to isolate pairs with a maximum whole-genome distance of 100 wgSNPs. Each point represents a pairwise comparison between two isolates. Blue lines indicate linear regression fits, and Pearson correlation coefficients (r) are shown in each panel.


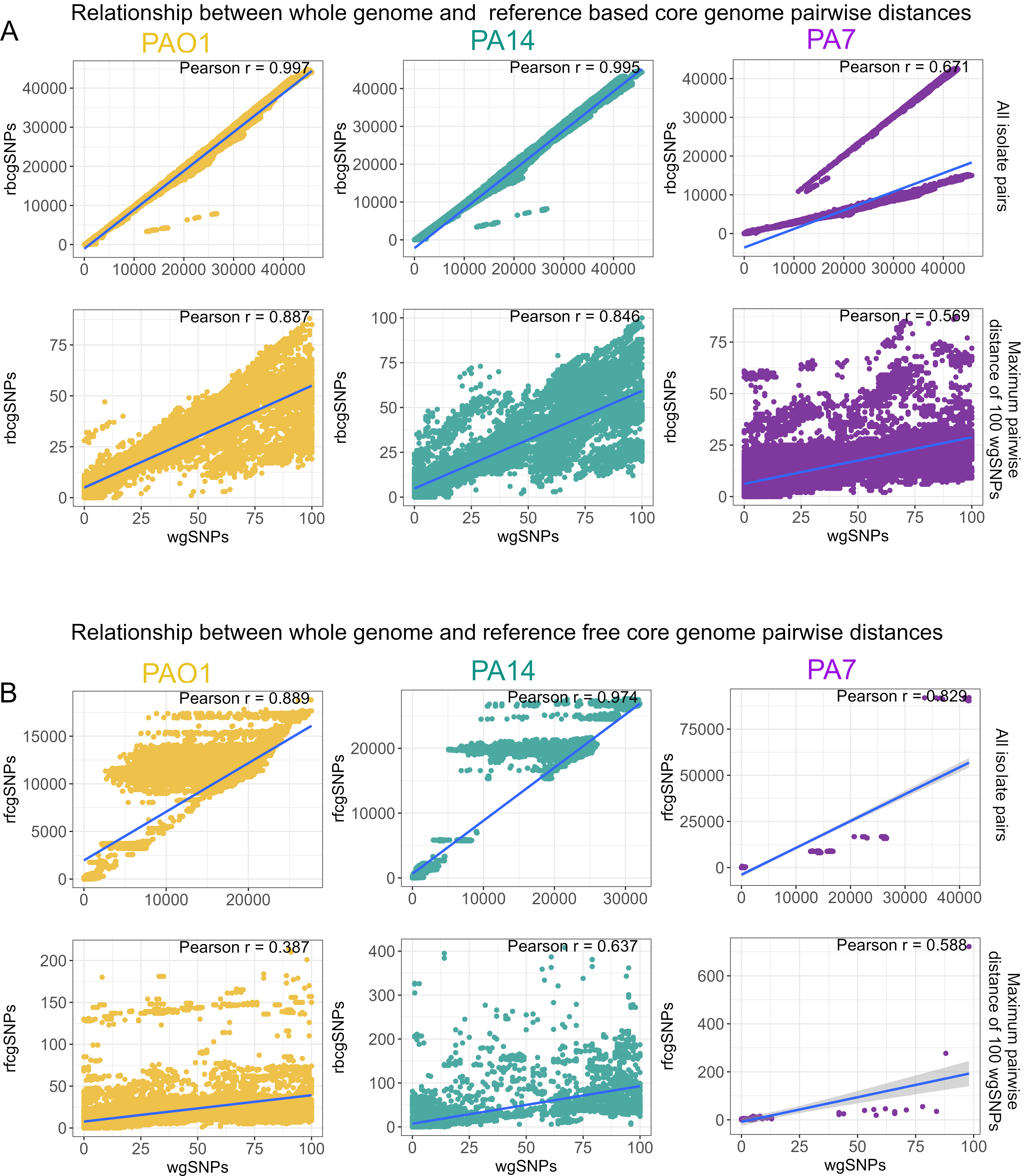


**Fig. S5. Genetic distance and epidemiological linkage for patient–patient and environment–patient isolate pairs.**
Contingency tables summarizing the relationship between genetic relatedness and epidemiological linkage score (ELS) for patient–patient (top) and environment–patient (bottom) isolate pairs. Genetic relatedness is classified according to the whole-genome SNP threshold (GT) of 24 SNPs, defining pairs as below or above the genetic threshold. ELS categories are shown as high, medium, low, or unlinked (UL). For each comparison type, the main tables report absolute counts of isolate pairs across GT and ELS categories. Additional panels show pooled linked versus unlinked pairs, as well as percentages stratified by ELS and by genetic threshold.


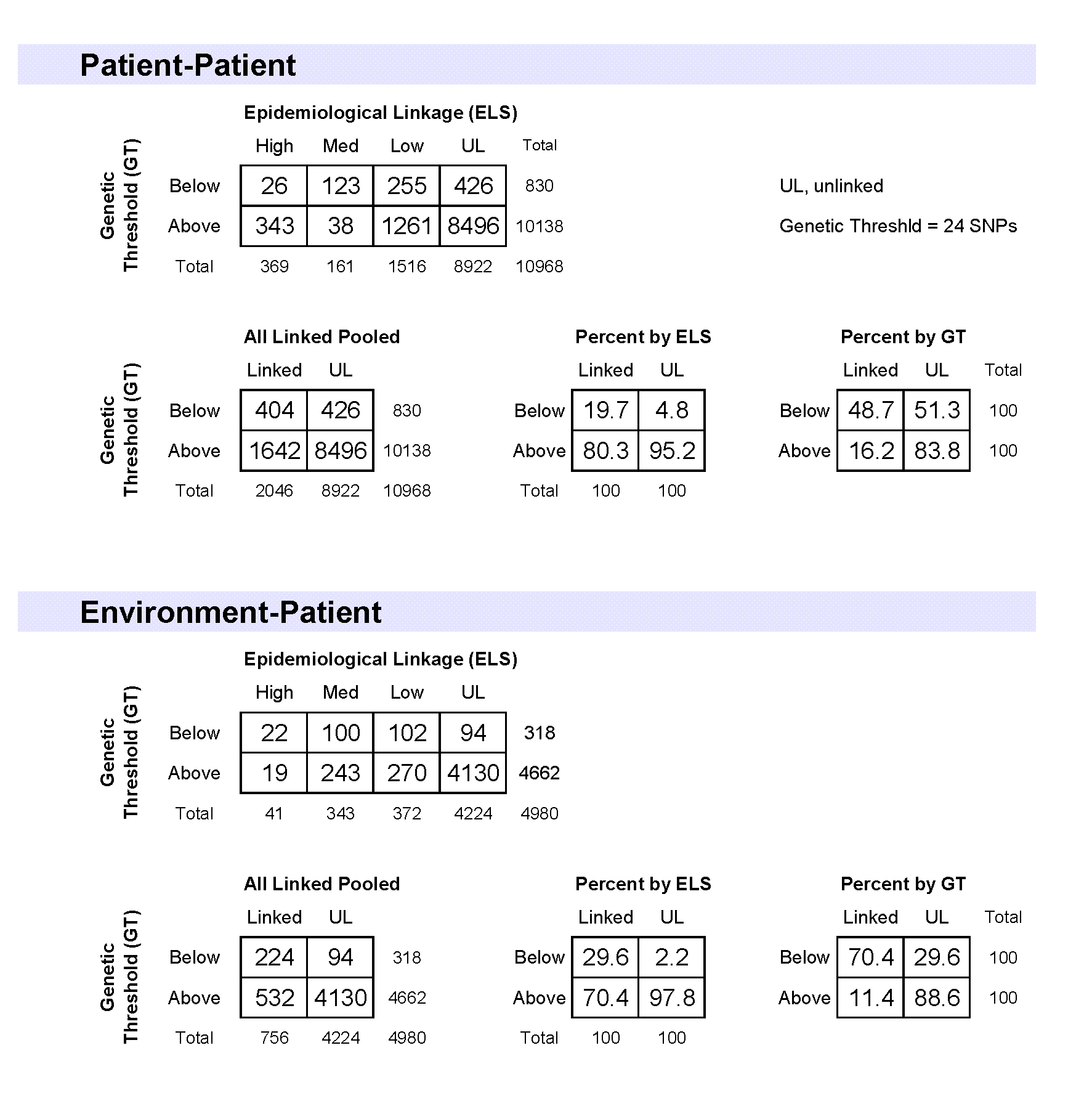


**Fig. S6. Composition of genomic clusters defined by a 24-SNP threshold.**
Network visualization of genomic clusters generated using a whole-genome SNP distance threshold of 24 SNPs. Each node represents an individual isolate, and clusters group isolates whose pairwise distances fall below the threshold. Nodes are colored by source, with clinical isolates shown in red and environmental isolates shown in blue.


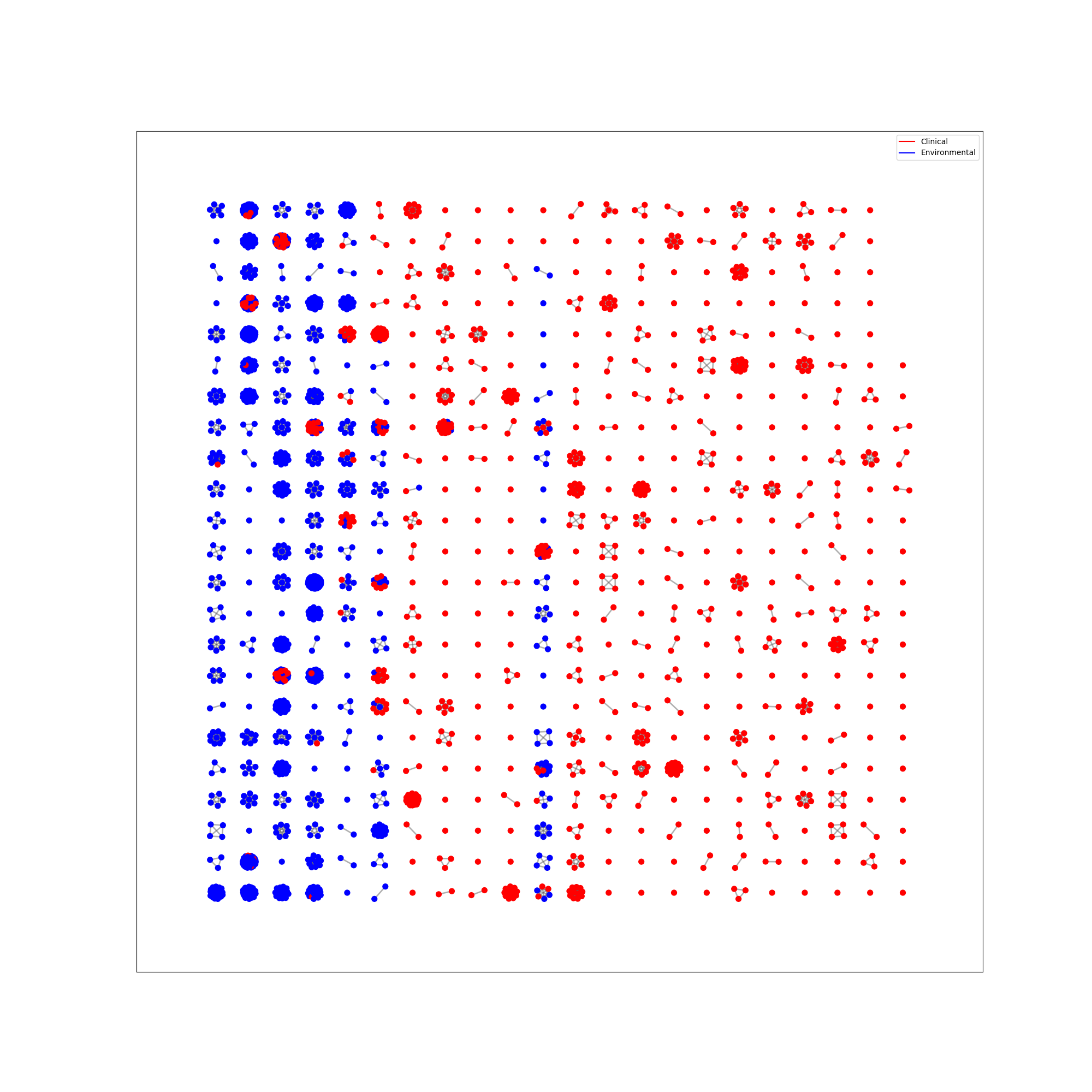


**Fig. S7. Source and patient/admission composition of genomic clusters.**
Sunburst plot summarizing the composition of genomic clusters defined using a 24-SNP whole-genome threshold. The inner circle represents all identified clusters. The first ring classifies clusters by source composition: clusters containing only clinical isolates, only environmental isolates, or both sources. The outer ring further subdivides clusters according to the number of patients involved (single patient vs. multiple patients) and the number of admissions per patient (single vs. multiple admissions).


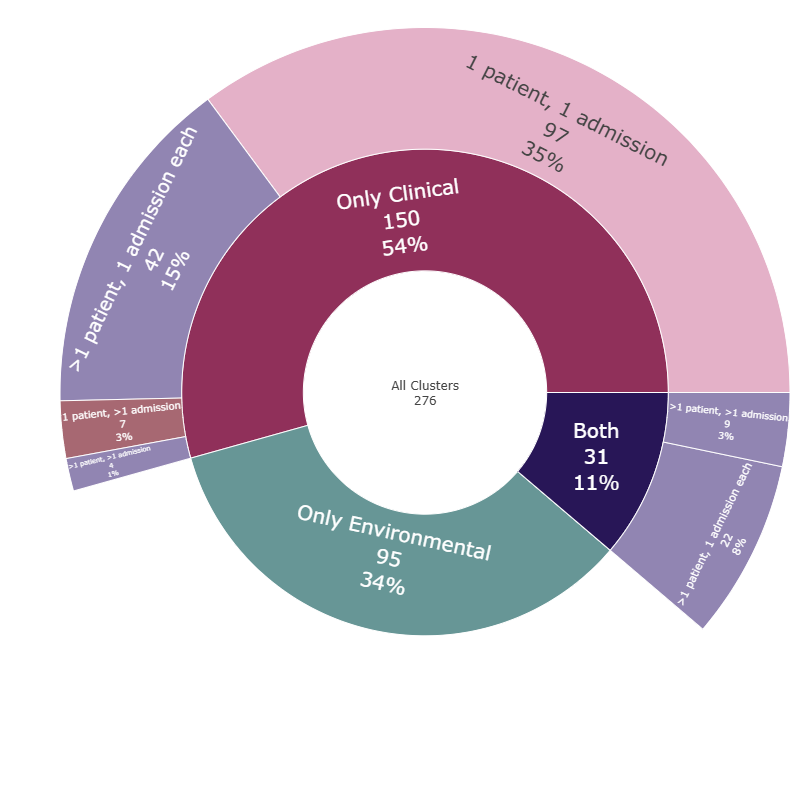


**Fig. S8. Genomic clusters involving isolates from multiple ICUs.**
Network representations of genomic clusters defined using a whole-genome SNP distance threshold of 24 SNPs, restricted to clusters that include isolates from more than one ICU. Nodes are colored according to ICU of origin (H, J, L, M, N, S, and T), as indicated in the legend.


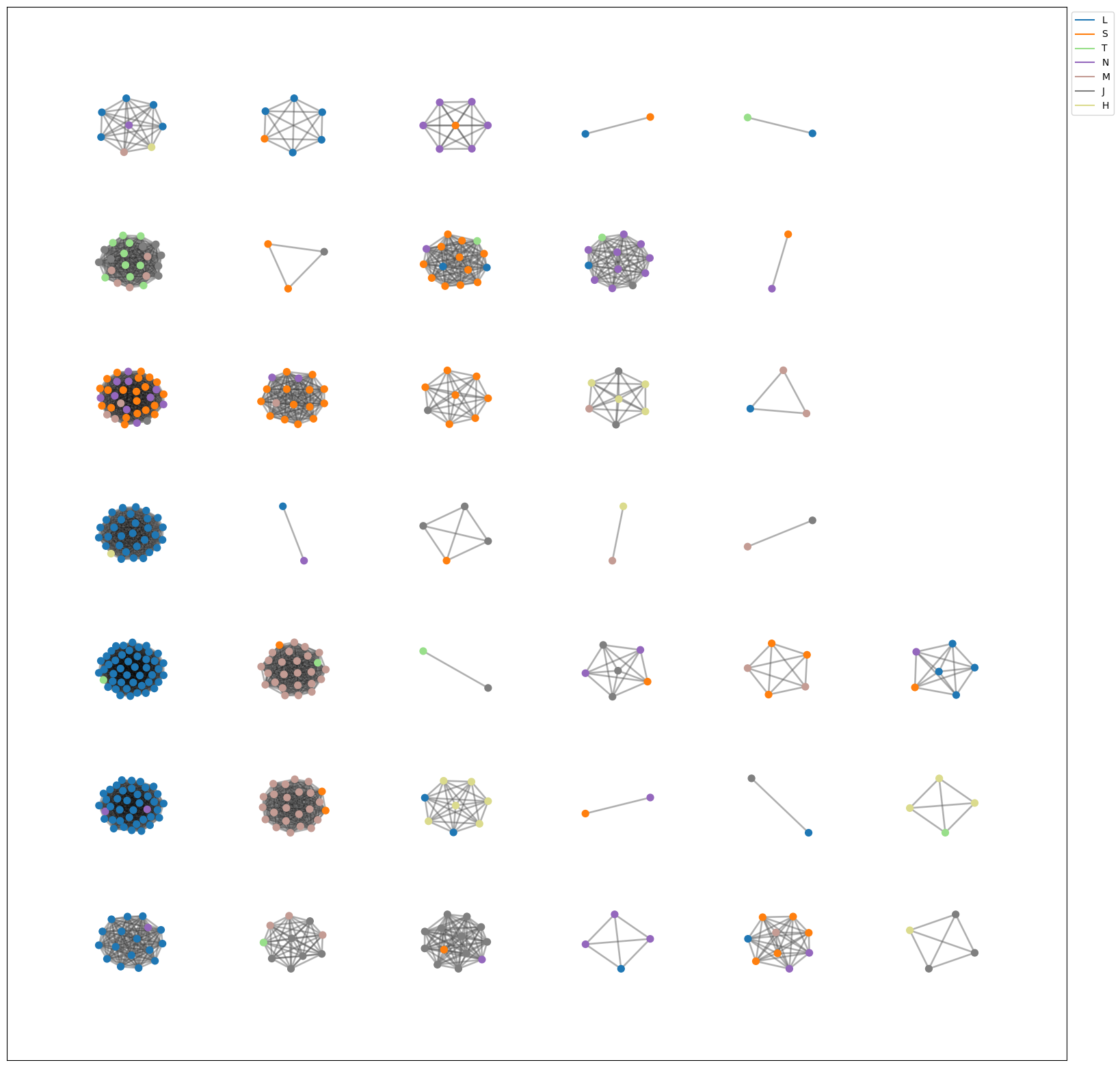


**Fig. S9. Genomic distance versus time by epidemiological linkage**

Scatter plots showing pairwise whole-genome SNP distances (wgSNPs) as a function of the number of days between isolate collection dates. Panels are stratified by epidemiological linkage score (ELS) categories (High, Medium, Low, and Not-linked) and by specific epidemiological relationships (Same Patient and Same Room). Each point represents a pairwise comparison between two isolates.


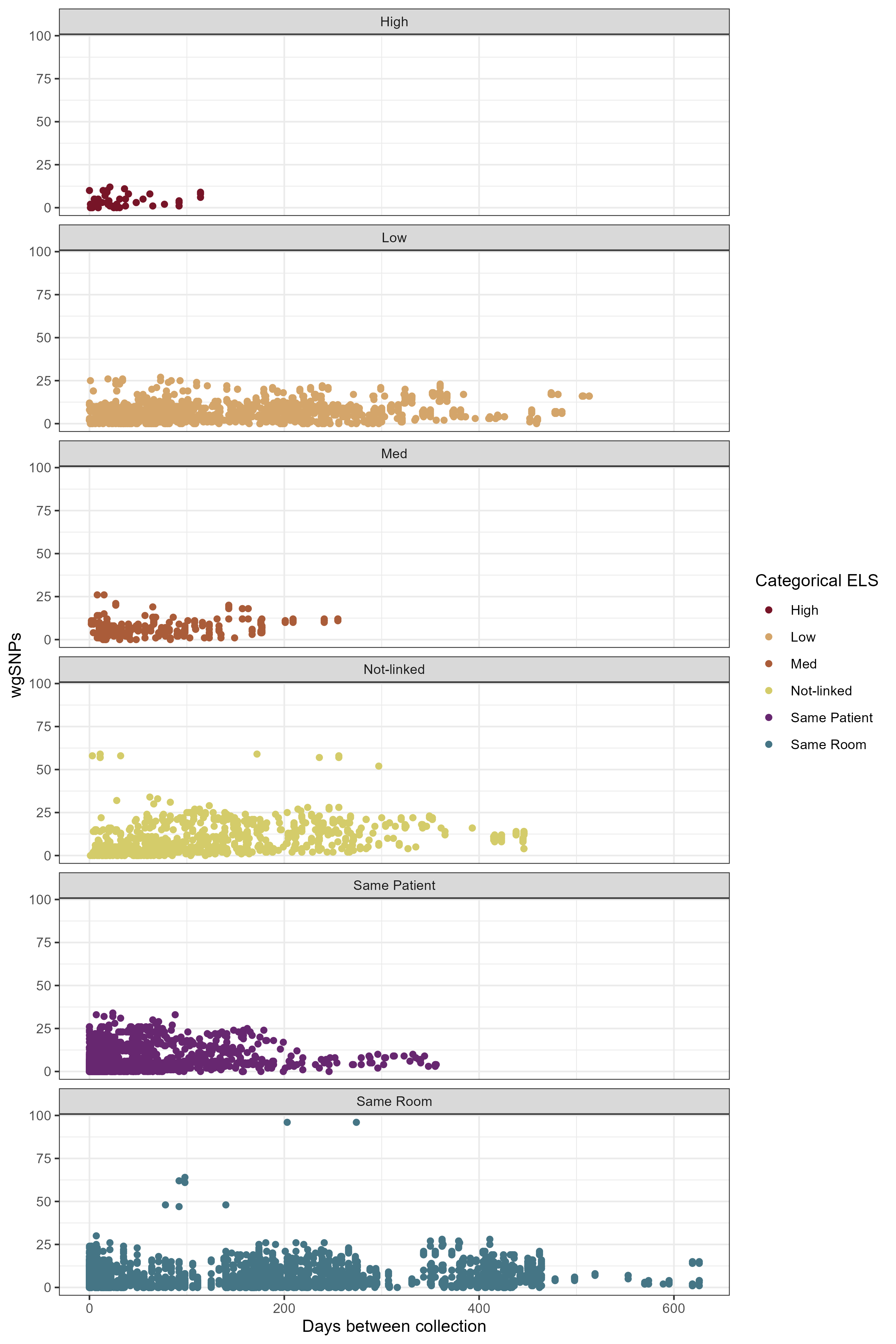

Supplement: Supplementary file 1 — Additional file 1. Contains all supplemental figures and figure legends. [file 13059_2026_4005_MOESM1_ESM.docx]
